# Dietary restriction involves NAD^+^-dependent mechanisms and a shift toward oxidative metabolism

**DOI:** 10.1111/acel.12273

**Published:** 2014-09-25

**Authors:** Natalie Moroz, Juan J Carmona, Edward Anderson, Anne C Hart, David A Sinclair, T Keith Blackwell

**Affiliations:** 1 Department of Genetics and Complex Diseases, Division of Biological Sciences, Harvard School of Public HealthBoston, MA, USA; 2 Section on Islet Cell and Regenerative Biology, Joslin Diabetes Center, Department of Genetics, Harvard Medical School, Harvard Stem Cell InstituteBoston, MA, USA; 3 Laboratory of Environmental Epigenetics and Program in Quantitative Genomics, Department of Environmental Health, Harvard School of Public HealthBoston, MA, USA; 4 Department of Neuroscience, Brown UniversityProvidence, RI, USA; 5 Department of Genetics and Paul F. Glenn Labs for the Biological Mechanism of Aging, Harvard Medical SchoolBoston, MA, USA; 6 Department of Pharmacology, School of Medical Sciences, The University of New South WalesSydney, NSW, Australia

**Keywords:** aging, dietary restriction, *C*.* elegans*, stress response, sirtuins, NAD^+^

## Abstract

Interventions that slow aging and prevent chronic disease may come from an understanding of how dietary restriction (DR) increases lifespan. Mechanisms proposed to mediate DR longevity include reduced mTOR signaling, activation of the NAD^+^-dependent deacylases known as sirtuins, and increases in NAD^+^ that derive from higher levels of respiration. Here, we explored these hypotheses in *Caenorhabditis elegans* using a new liquid feeding protocol. DR lifespan extension depended upon a group of regulators that are involved in stress responses and mTOR signaling, and have been implicated in DR by some other regimens [DAF-16 (FOXO), SKN-1 (Nrf1/2/3), PHA-4 (FOXA), AAK-2 (AMPK)]. Complete DR lifespan extension required the sirtuin SIR-2.1 (SIRT1), the involvement of which in DR has been debated. The nicotinamidase PNC-1, a key NAD^+^ salvage pathway component, was largely required for DR to increase lifespan but not two healthspan indicators: movement and stress resistance. Independently of *pnc-1*, DR increased the proportion of respiration that is coupled to ATP production but, surprisingly, reduced overall oxygen consumption. We conclude that stress response and NAD^+^-dependent mechanisms are each critical for DR lifespan extension, although some healthspan benefits do not require NAD^+^ salvage. Under DR conditions, NAD^+^-dependent processes may be supported by a DR-induced shift toward oxidative metabolism rather than an increase in total respiration.

## Introduction

The reduction of food consumption without malnutrition, termed dietary restriction (DR), is the most conserved intervention known to increase lifespan. DR promotes longevity in essentially all eukaryotes, including yeast, rotifers, spiders, *Drosophila*,*C. elegans*, many strains of mice, and possibly non-human primates (Fontana *et al*., 2010; Haigis & Sinclair, 2010; Guarente, 2013; Colman *et al*., 2014). In mammals, DR improves many physiological parameters that are associated with aging, and delays aging-associated disorders ranging from neurodegenerative disease to cancer. An understanding of how DR confers these benefits may allow development of interventions that mimic the effect of DR, but circumvent the difficulties of severely reducing nutrient intake.

The response to DR is thought to be an evolutionary adaptation to survive periods of low food availability, predicting that mechanisms through which DR extends lifespan are likely to be conserved. Indeed, genetic analyses of the model organisms *S. cerevisiae*,*D. melanogaster,* and *C. elegans* have implicated essential nutrient-sensing mechanisms in the benefits of DR. Considerable evidence indicates that DR acts on the nutrient-sensing mechanistic target of rapamycin complex 1 (mTORC1) kinase, which is activated by amino acid availability, oxygen, and growth signaling (Fontana *et al*., 2010; Johnson *et al*., 2013). Lower levels of mTORC1 activity, as would be expected to be encountered in DR, result in reduced protein and lipid synthesis, increased autophagy and stress-defense activity, enhanced regenerative capacity, and in longer life in various organisms.

Sirtuins, which modulate transcription and numerous metabolic processes, comprise another set of energy-sensing mechanisms involved in DR (Haigis & Sinclair, 2010; Guarente, 2013). Sirtuins are protein deacylases or ADP-ribosyltransferases that convert nicotinamide adenine dinucleotide (NAD^+^) into nicotinamide (Nam), which in turn inhibits sirtuins. It has been proposed that DR promotes health and longevity by activating the nuclear sirtuin Sir2 (SIRT1 in mammals) (Haigis & Sinclair, 2010; Guarente, 2013). In mammals, SIRT1 and other sirtuins mediate DR effects on parameters such as mitochondrial biogenesis, metabolism, body weight, and glucose tolerance, and in mice overexpression of SIRT1 in the brain increases lifespan (Haigis & Sinclair, 2010; Guarente, 2013; Satoh *et al*., 2013). While these data suggest a major role for sirtuins in DR, the involvement of sirtuins in DR lifespan extension has remained a subject of debate. In lower organisms, the requirement for SIRT1 for DR longevity depends upon the experimental conditions, and in *C. elegans,* the SIRT1 ortholog *sir-2.1* was required for lifespan extension by a weak mutation in *eat-2*, a genetic DR model, but not in other *eat-2* experiments or DR methods that utilize bacterial food dilution (Mair *et al*., 2009; Kenyon, 2010; Burnett *et al*., 2011; Guarente, 2013).

From yeast to mammals, sirtuin activity is increased by conditions that increase NAD^+^ availability or reduce Nam levels (Haigis & Sinclair, 2010; Guarente, 2013). Interventions that boost NAD^+^ levels increase *C. elegans* lifespan and restore mitochondrial function in aging mice, in each case dependent upon *sir-2.1/SIRT1* (Gomes *et al*., 2013; Mouchiroud *et al*., 2013b). *C. elegans* lifespan was also increased in a *sir-2.1*-dependent manner by reduced activity of PARPs (poly (ADP-ribose) polymerases, PARPs), which consume NAD^+^ (Mouchiroud *et al*., 2013a). Together, these findings support the idea that higher NAD^+^ levels promote longevity by activating SIR-2.1/SIRT1. In yeast, DR longevity requires the nicotinamidase Pnc1, which acts in a salvage pathway that allows Nam generated by sirtuins and other NAD^+^ consumers to be recycled to NAD^+^ (Fig. S1, Supporting Information) (Anderson *et al*., 2003). The importance of NAD^+^ salvage in DR has not been assessed in a metazoan, but overexpression of the PNC-1 ortholog D-NAAM increases *Drosophila* lifespan in a Sir2-dependent manner (Balan *et al*., 2008). In yeast, DR shifts metabolism away from glycolysis and toward respiration, which generates NAD^+^ by consuming NADH (Guarente, 2013; Schleit *et al*., 2013). While DR reduces caloric availability, it has been proposed that DR paradoxically increases respiration by inducing this metabolic shift, leading to higher NAD^+^ levels that drive lifespan extension (Bishop & Guarente, 2007; Guarente, 2013).

*Caenorhabditis elegans* provides a powerful metazoan model for studying DR because it has a short lifespan and is amenable to genetic disruption of processes that might be essential in mammals. The genetic requirements for lifespan extension vary among *C. elegans* DR regimens (Greer & Brunet, 2009; Mair *et al*., 2009). This variation may arise because of differences in culture conditions, including differences between liquid and solid culture protocols with respect to oxygen exposure and the extent to which the animals move. This diversity has allowed identification of a wider range of DR-associated mechanisms than would be possible with one ‘standard’ protocol (Greer & Brunet, 2009; Mair *et al*., 2009). Here, we investigated requirements for stress defense and NAD^+^-associated mechanisms, using a new liquid feeding DR method that robustly extends lifespan and minimizes maintenance. DR lifespan extension depended upon stress-defense regulators that are regulated by mTOR and growth pathways, and also upon SIR-2.1. The NAD^+^ salvage pathway enzyme PNC-1 was required for DR lifespan extension but not some healthspan benefits, providing the first evidence in a metazoan that implicates NAD^+^ salvage in DR. Independently of *pnc-1*, DR reduced total oxygen consumption but increased the proportion of respiration devoted to ATP production. Apparently, DR drives the activity of key NAD^+^-associated mechanisms through a shift toward oxidative metabolism, but does not necessarily increase overall respiration rates.

## Results

### A simple liquid protocol for *C. elegans* dietary restriction

Among the approaches used to restrict *C. elegans* dietary intake, liquid DR protocols (Table1, Table S1, Supporting Information) provide the advantage of allowing for: (i) variation of bacterial food availability, (ii) monitoring of food concentration over time, (iii) utilization of standard *C. elegans* strains, and (iv) scaling up for biochemical analyses. As *C. elegans,* liquid DR methods are generally labor intensive, we developed a protocol that requires relatively low maintenance (Fig.1A). On day one of adulthood, worms are transferred to NGM plates seeded with bacteria that have been maintained in antibiotics for 1 week at 4 °C (treated OP50). On day three of adulthood, these worms are transferred to liquid cultures that contain treated OP50 at different concentrations (Fig.1A). The treatment step prevents bacteria from re-entering growth phase (Fig. S2), eliminating the need for frequent food replacement.

**Table 1:** Comparison of *C. elegans* liquid DR methods

Liquid DR method	Age at DR onset	Food source	Antibiotics present	Average lifespan of DR animals (days)	Average DR lifespan extension for WT at 20 °C (%)	Epistasis analysis
Klass^a^	48 hr. post-hatching	OP50	None	26	73	NA
Vanfleteren^b^	L4	E. coli 9001	None, 50uM FUdR	12*	140^τ^*	*daf-2* independent, *daf-16* partially required
Dillin^c^^d^	Day-2 adult	OP50	50 µg mL^−1^ Carb, 1 µg mL^−1^ Tet, 10 µg mL^−1^ Kan, 100 µg mL^−1^ FUdR	42	60 (26 in *smg–1* ts)^γ^	*pha-4* required in *smg-1* ts, *daf-16* partially required, *aak-2* not required, *sir-2.1;sir-2.3* not required
Brunet^e^	Day-2 adult	OP50-1	50 µg mL^−1^ Amp, 1 µg mL Tet, 10 µg mL^−1^ Kan, 100 mg L^−1^ FUdR	42†	51†	*Requirement for aak-2* and *daf-16*
Guarente^f^	L4/young adult	HT115	1 mg mL^−1^ Erythro, 50 µg mL^−1^ Amp, 12.5 µg mL^−1^ FUdR, 1mM IPTG	33	22	*skn-1* required
Sinclair, Hart, Blackwell	Day-3 adult	OP50	50 µg mL^−1^ Amp, 10 µg mL^−1^ Kan, 1 µg mL^−1^ Tet, NYS, 100 µg mL^−1^ FUdR	39 (42 DD in WT) (39 DD in smg–1 ts)	60 (76 DD in WT) (45 DD in *smg-1* ts)	See figures

This table compares our DR method with other *C. elegans* liquid DR methods.

a Klass (1977)).

b Houthoofd *et al*. (2007).

c Panowski *et al*. (2007).

d Mair *et al*. (2009).

e Greer & Brunet (2009).

f Bishop & Guarente (2007).

* Values approximated from graph.

† Average calculated from all presented data. τ Animals were grown at 17 °C from hatching, at L4 were switched to 24 °C for remainder of lifespan. γ animals were grown at 25 °C from hatching, at first day of adulthood switched to 20 °C for 1 day, then 15 °C for remained of lifespan.

**Figure 1:**
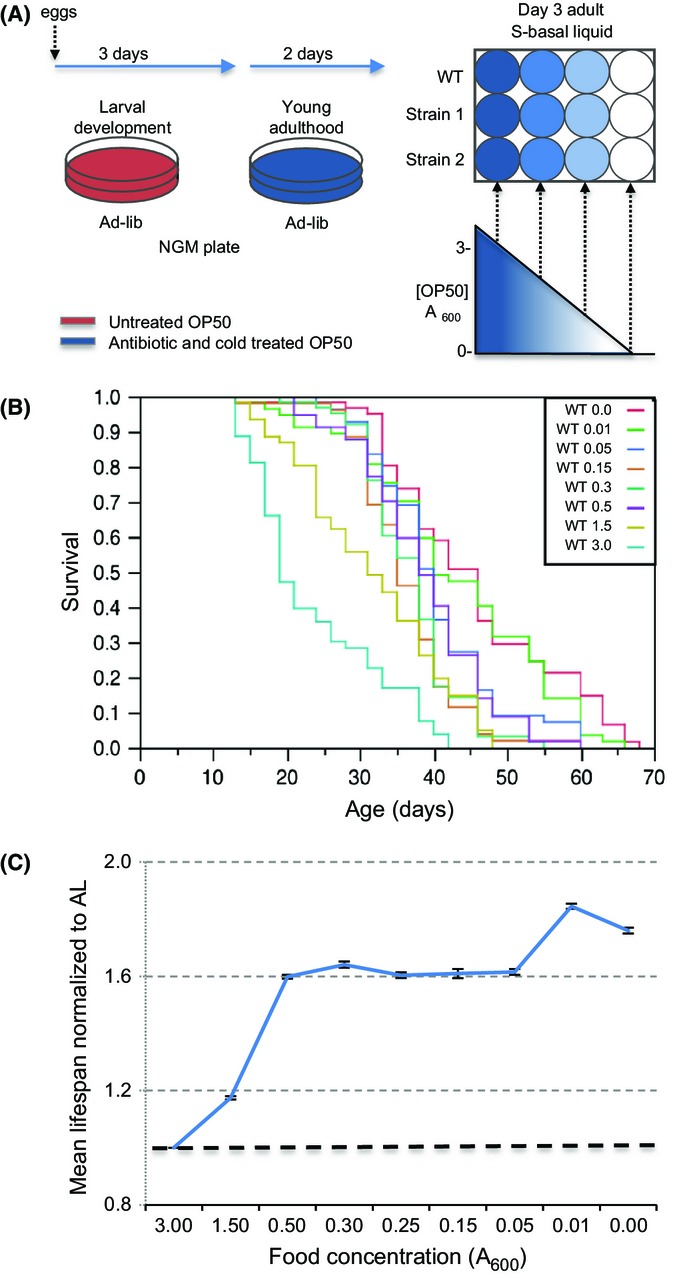
Lifespan extension from liquid-based DR and DD. (A) Description of the liquid DR method*. C. elegans* were allowed to develop under standard *C. elegans* conditions, then moved to NGM plates seeded with treated bacterial food (strain OP50) as day-one adults. These animals were moved to 12-well plates containing 2.5 mL of different concentrations of treated OP50 after 2 days of AL feeding, then moved to fresh cultures every 2–3 weeks. (B) Representative lifespans of wild-type (N2) worms that were fed different concentrations of treated bacteria (indicated as bacterial OD) and examined in parallel. Mean values and analysis of this experiment are presented in Table S12 (experiment 1C). (C) Effect of food concentration on mean lifespan. This graph describes a composite of 21 independent experiments, each of which examined a range of serially diluted treated bacteria in parallel. Lifespans were normalized to AL by dividing the mean lifespan at each food concentration by the AL value determined in parallel. DR and DD increased lifespan by 59.4% and 76%, respectively. SEM is shown. Compared to AL, a two-tailed *t-*test for all A_600_ values, *P *< 0.001. Individual experiments are presented in Table S12.

To determine whether a gene is required for DR and does not simply alter the optimal response to DR, lifespan must be examined over a range of bacterial food concentrations (Mair *et al*., 2009). We designated 3 × 10^9^ C.F.U. mL^−1^ (3.0 A_600_) as *ad libitum* (AL) feeding, because at this concentration wild-type (WT) lifespan most closely resembled published observations on plates and in liquid (Fig.1B,C, Table S3). A plot of mean lifespan vs. food would be parabolic if concentrations below that optimal for DR resulted in starvation-like effects (Mair *et al*., 2009). However, combined analyses of wild-type (WT) worms resulted in a plateau-shaped plot that exhibited bimodal lifespan maxima (Fig.1B,C; Table S3). Even complete removal from food increased lifespan robustly, as was seen in dietary deprivation (DD) protocols in which *C. elegans* adults were maintained on plates without food beginning at day 2 (Kaeberlein *et al*., 2006; Lee *et al*., 2006). By initially keeping adults on plates with food for 3 days, longer than other liquid protocols (Table1), we apparently made it possible for the animals to live longer even if they subsequently relied only on stored nutrients. We refer to the first local maximum on the lifespan extension curve as DR. This occurred at 5 × 10^8^ C.F.U. mL^−1^ (0.5 A_600_), a six-fold reduction in food compared to AL (Fig.1B,C, Table S3). The second local maximum occurred at 1 × 10^7^ C.F.U. mL^−1^ (0.01 A_600_) (Fig.1B,C, Table S3), which is close to complete DD. Over the range of bacteria concentrations, our regimen increased mean lifespan by 61–84.5%, (Fig.1C, Table S3), one of the largest extensions seen in *C. elegans* DR (Table1).

### Genetic analysis of DR-associated mechanisms

We compared our protocol to other methods by investigating whether lifespan extension required a set of regulators that were needed in some but not necessarily all *C. elegans* DR protocols in which they were examined. We first looked at the transcription factors DAF-16/FOXO, SKN-1/Nrf 1,2,3, and PHA-4/FOXA, each of which is regulated by mTORC1 signaling (Fig. S4) (Johnson *et al*., 2013). DAF-16/FOXO is required for longevity from reduced activity of insulin/IGF-1 signaling (IIS) or mTORC1 (Fig. S4) (Kenyon, 2010; Robida-Stubbs *et al*., 2012). DAF-16 is completely or partially required for lifespan extension by some DR protocols (Greer *et al*., 2007; Greer & Brunet, 2009; Honjoh *et al*., 2009), but appears to be dispensable in other DR regimens (summarized in Greer & Brunet, 2009). Our data are consistent with the former view: a lack of DAF-16 substantially reduced lifespan extension from either DR (from 58.2% to 26.3%) or DD (from 93.4% to 39%) (Fig.2A,B, Table S4). SKN-1/Nrf has important functions in stress and starvation responses, proteostasis, and metabolism, and contributes to lifespan extension from downregulation of either IIS or mTORC1 (Fig. S4) (Tullet *et al*., 2008; Robida-Stubbs *et al*., 2012; Glover-Cutter *et al*., 2013). *skn-1* was required for longevity in a liquid DR protocol that was less robust than this one, but not in a plate DR regimen (Table1) (Bishop & Guarente, 2007; Greer & Brunet, 2009). Here, lack of *skn-1* greatly impaired the lifespan increase from DR (from 48.3% to 20.9%) or DD (from 91% to 13.3%) (Fig.2C,D, Table S5). PHA-4/FOXA is involved in autophagy and is required for lifespan extension from reduced TOR activity, the genetic DR model *eat-2*, and a liquid DR method (Panowski *et al*., 2007; Sheaffer *et al*., 2008), although it was not required in a DR protocol that involved solid media (Greer & Brunet, 2009). *pha-4* was needed for DR lifespan extension in our protocol, although DR was less effective in the *smg-1(ts)* background in which the *pha-4* mutation is maintained (Panowski *et al*., 2007; Sheaffer *et al*., 2008) (23.2% lifespan increase in *smg-1(ts)* vs. 77.3% in WT from DR, and 44.6% vs. 77.3% from DD) (Fig.2E,F, Table S6).

**Figure 2:**
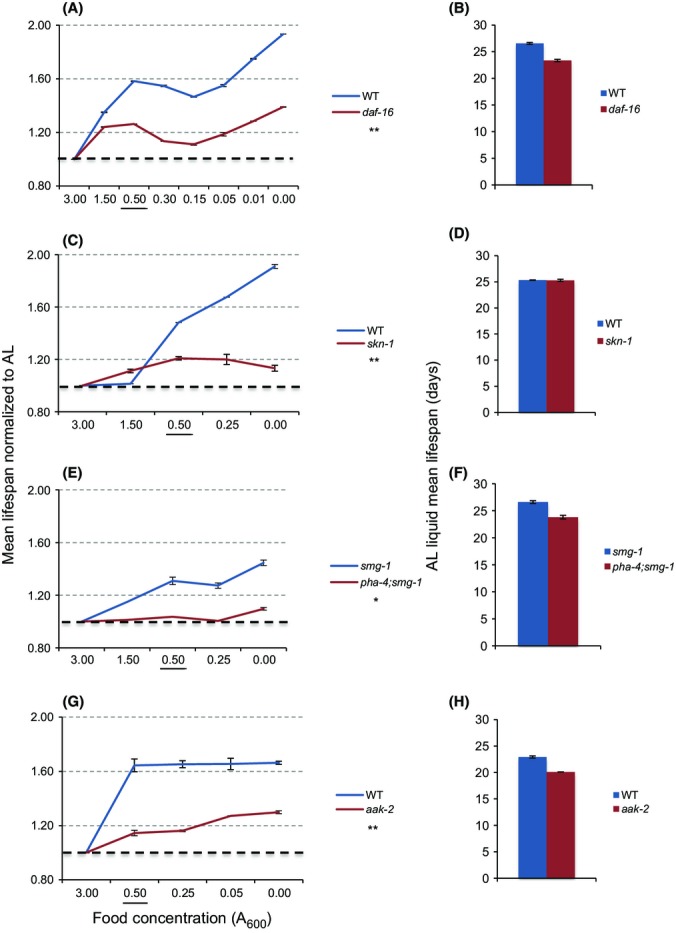
Importance of stress response mechanisms for DR lifespan extension. (A) Lack of DAF-16 impaired DR lifespan extension. AL lifespans obtained in parallel are shown in (B). (C,D) *skn-1* was required for DR to extend lifespan. Whereas WT worms experienced an average of 48.3% and 91.0% increase in lifespan upon DR and DD, respectively, these increases were only 20.9% and 13.3% in predicted null *skn-1* mutants. (D) Under AL liquid conditions, *skn-1* mutants’ mean lifespan was equal to that of WT, in contrast to results obtained on plates (Tullet *et al*., 2008). (E, F) *pha-4(zu225)* mutation eliminated DR longevity in the control *smg-1(cc546ts)* background. *smg-1* lifespan was increased 33.2% by DR and 38.8% by DD, respectively. (G,H) The AMPK subunit AAK-2 is required for full DR and DD lifespan extension. Composites of all analyses are shown, with individual experiments presented in Table S13 (Fig.2A,B), Table S14 (Fig.2C,D), Table S15 (Fig.2E,F), and Table S16 (Fig.2G,H). Two-way ANOVA analysis across the bacterial gradient (compared to control): *P = 0.0004, **P = 0.0001. The food concentration 0.50 A_600_ is underlined to facilitate comparison of results.

We also looked at the 5′ AMP-activated kinase (AMPK), which coordinates an adaptive response to low-energy availability that enhances oxidative metabolism, mitochondrial biogenesis, and autophagy, and is inhibited by mTORC1 in mammals (Mair *et al*., 2011). Increased activity of the AMPK catalytic subunit AAK-2 increases *C. elegans* lifespan (Apfeld *et al*., 2004; Mair *et al*., 2011) by inhibiting the transcriptional co-activator CRTC (Mair *et al*., 2011) and activating DAF-16/FOXO (Greer *et al*., 2007; Greer & Brunet, 2009). AMPK was important for DR lifespan extension in *Drosophila* (Stenesen *et al*., 2013), and in *C. elegans* was required in some DR methods but not others (Greer *et al*., 2007; Greer & Brunet, 2009; Mair *et al*., 2009). In our system, *aak-2* loss reduced lifespan extension from DR (from 64.4% to 14.5%) or DD (From 66.4% to 30%) (Fig.2G,H, Table S7).

### Importance of SIR-2.1 and NAD^+^ salvage in DR

SIRT1 has been implicated in DR in various organisms, but was required for DR lifespan extension in only one of several previous *C. elegans* studies (Introduction; Table1). SIRT1/SIR-2.1 functions both upstream and downstream of AMPK and activate DAF-16/FOXO (Canto *et al*., 2009; Rizki *et al*., 2011; Price *et al*., 2012; Guarente, 2013; Mouchiroud *et al*., 2013a), suggesting that it might be important in our DR regimen. Accordingly, a null *sir-2.1* mutation significantly reduced lifespan extension from either DR (from 69.5% to 40.5%) or DD (From 89.4% to 47.4%) (Fig.3A,B, Table S8). The importance of *sir-2.1* for DR-associated longevity was particularly striking because the *sir-2.1* mutant lived slightly longer than WT under AL conditions (Fig.3B, Table S8).

**Figure 3:**
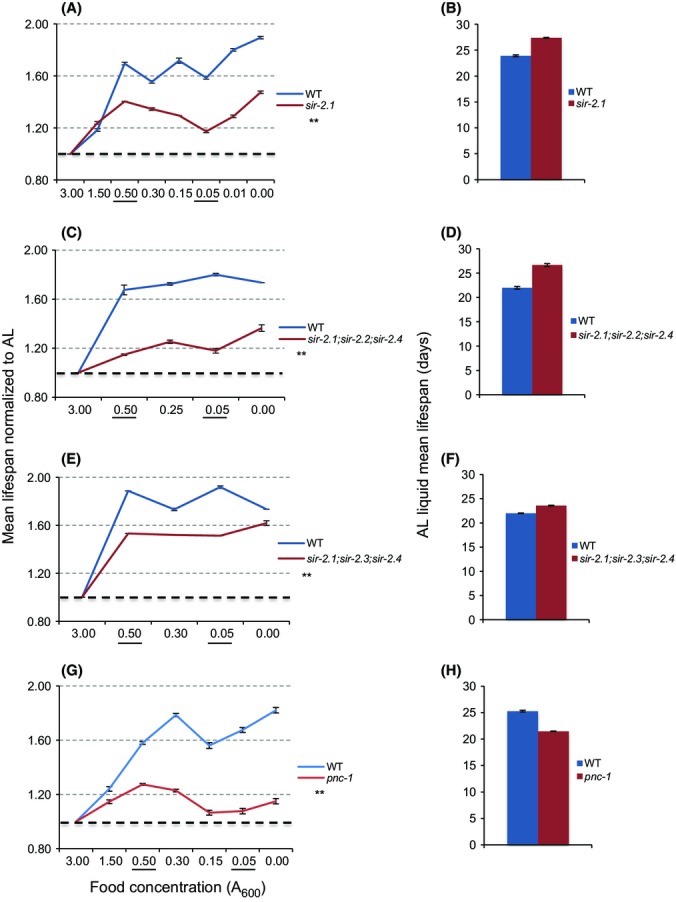
The NAD^+^ salvage pathway and NAD^+^-dependent SIRT1/*sir-2.1* regulate DR lifespan. (A, B) Reduced DR response in *sir-2.1(ok434)* mutants. Note that the *sir-2.1* mutants live longer than WT worms under AL feeding. (C-F) Impairment of DR lifespan extension in *sir-2.1;sir-2.2;sir-2.4* (C, D) and *sir-2.1;sir-2.3;sir-2.4* (E, F) triple mutants. (G, H) *pnc-1* is required for DR lifespan extension. Composites are shown, with data from individual experiments presented in Table S17 (Fig.3A,B), Table S18 (Fig.3C,D), Table S19 (Fig.3E,F), and Table S20 (Fig.3G,H). Two-way ANOVA analysis: **P = 0.0001.

We considered the possibility that the three additional *C. elegans* sirtuin genes might have DR-related functions that overlap with those of *sir-2.1*. *sir-2.2* and *sir-2.3* are nearly identical orthologs of the mitochondrial sirtuin SIRT4, and *sir-2.4* is orthologous to the nuclear sirtuins SIRT6 and SIRT7. Each of these sirtuins modulates critical metabolic processes (Haigis & Sinclair, 2010), and in *C. elegans,* SIR-2.4 acts non-catalytically to promote DAF-16 activity under stress conditions (Chiang *et al*., 2012). As *sir-2.2* and *sir-2.3* are located less than one kilobase apart (wormbase.org), making it difficult to disrupt them simultaneously, we analyzed the triple-null mutants *sir-2.1; sir-2.2; sir-2.4* and *sir-2.1; sir-2.3; sir-2.4*. Each of these mutants responded to DR and DD comparably to *sir-2.1* (Fig.3C–F, Tables S9 and S10). The data do not reveal any additive requirement for these sirtuins and *sir-2.1*, but do not exclude the possibility that redundancy might mask a role for *sir-2.2* and *sir-2.3*.

The importance of *sir-2.1* for DR lifespan extension predicts that NAD^+^ availability would be a critical factor. Moreover, NAD^+^ has critical sirtuin-independent functions that could be important in DR (Pollak *et al*., 2007). NAD^+^ serves as a co-enzyme in numerous metabolic electron transfer reactions, and its reduced form NADH plays a central role in mitochondrial electron transport. NAD^+^ is also the precursor to NADP^+^ (NAD phosphate), the reduced form of which (NADPH) is essential for cellular oxidative defense and other reductive detoxification reactions (Pollak *et al*., 2007). We investigated the role of NAD^+^ in DR by examining requirements for PNC-1, the *C. elegans* ortholog of the NAD^+^ salvage nicotinamidase Pnc1 (Fig. S1) (Vrablik *et al*., 2009). A predicted null *pnc-1* mutation dramatically reduced the lifespan increase associated with either DR (from 57.2% to 32.1% at OD 0.5 and 77.2 to 27 at OD 0.3) or DD (from 82% to 15%) (Fig.3G,H, Table S11), suggesting that the NAD^+^ salvage pathway plays a major role in *C. elegans* DR.

In metazoa, DR not only extends lifespan but improves many parameters associated with health and resistance to chronic disease and slows their decline during aging (Fontana *et al*., 2010; Haigis & Sinclair, 2010; Guarente, 2013). This beneficial effect on ‘healthspan’ has been described not only in mammals but also in *C. elegans*, in which DR increases muscle activity (Greer *et al*., 2007). We investigated the importance of the NAD^+^ salvage pathway for muscle activity by comparing how DR affects movement in WT and *pnc-1* animals. In *pnc-1* mutants, body-wall muscle function is impaired (Vrablik *et al*., 2011), and under AL conditions their rate of spontaneous body bending was reduced compared to WT (Fig.4A, Table S21). Surprisingly, however, DR comparably increased the frequency of bending in older WT and *pnc-1* animals (Fig.4A,B, Table S21). As a second healthspan measure, we examined thermotolerance, which is characteristically increased by DR in *C. elegans* (Kenyon, 2010). In WT animals, heat resistance declined with age, but this decline was reversed by day 15 under DR (Fig.4C,D, Table S22). In *pnc-1* mutants, DR increased thermotolerance similarly (Fig.4C,D, Table S22). These improvements in healthspan parameters suggest that *pnc-1* mutants are not refractory to DR lifespan extension because they are simply sick. Apparently, in *C. elegans,* the NAD^+^ salvage pathway is required for DR to increase lifespan, but not to confer these healthspan benefits.

**Figure 4:**
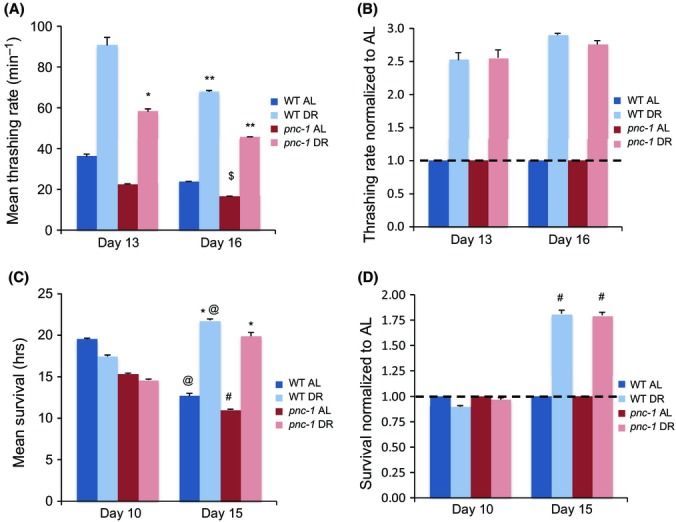
DR increases movement and stress resistance independently of NAD^+^ salvage. (A,B) DR increased the rate of spontaneous movement comparably in aging WT and *pnc-1(pk9605)* animals. Body bends per minute were scored. Note that *pnc-1* mutation did not affect the percentage increase associated with DR. (C,D) DR comparably increased thermotolerance (survival at 38 °C) in WT and *pnc-1* animals. The time points indicated refer to days after hatching. Composites of all analyses are shown, with individual experimental data, mean, standard error, percent change, and statistical analysis presented in Table S21 (Fig.4A,B), and Table S22 (Fig.4C,D). *t-test vs. AL,*P* < 0.05; **t-test vs. AL,*P* < 0.001; $*t*-test vs. age 13, *P* < 0.01; @*t*-test vs. age 10, *P* < 0.065; #*t*-test vs. age 10, *P* < 0.025.

### DR reduces total oxygen consumption but proportionally increases productive respiration

Another source of NAD^+^ is conversion from NADH during mitochondrial respiration. Based upon data from yeast, it has been proposed that DR elevates NAD^+^ levels by increasing respiration (Guarente, 2013). However, two *C. elegans* studies reached opposite conclusions with respect to DR effects on respiration, and an analysis of *Drosophila* did not see an increase in respiration with DR (Hulbert *et al*., 2004; Bishop & Guarente, 2007; Houthoofd *et al*., 2007). Previous *C. elegans* studies of DR measured oxygen consumption in liquid culture using the Clark electrode, which analyzes large samples that are difficult to generate in an age-matched fashion. We circumvented this issue by measuring oxygen consumption rate (OCR) using the Seahorse XF24 Analyzer, which can precisely examine tens of animals per replicate.

We investigated how aging, DR, and the NAD^+^ salvage pathway influence respiration by examining WT and *pnc-1* animals under AL and DR conditions at 9,12, and 16 days after hatching (3, 6, and 9 days of DR). Under AL conditions, the OCR per worm decreased with age in WT animals (Fig.5A, Tables S23 and S24), in close agreement with a recent study that used the XF24 (Mouchiroud *et al*., 2013b). *C. elegans* shrink in size post-reproductively, however, and with age exhibit profound sarcopenia in pharyngeal and body-wall muscles (Herndon *et al*., 2002). Respiration was therefore modestly elevated in older animals on a per-protein mass basis (Day 16; Fig.5B, Tables S25 and S26). Surprisingly, DR markedly reduced the OCR per worm at each day examined on either a per-worm or per-mass basis (Fig.5C,D, Tables S27 and S28). Similar trends were apparent in *pnc-1* mutants. Under our conditions, therefore, DR lowered overall oxygen consumption, and the NAD^+^ salvage pathway was not required to maintain respiration rates during DR.

**Figure 5:**
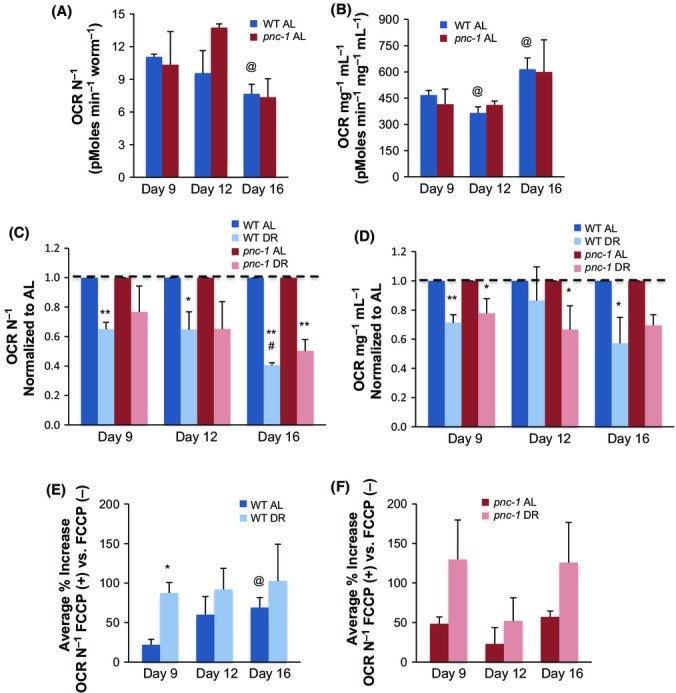
Differential effects of DR on overall and productive respiration. (A) Oxygen consumption per animal decreases with age. One-way ANOVA WT day 9 vs. day 12 vs. day 16, *P* < 0.0001, one-way ANOVA *pnc-1* day 9 vs. day 12 vs. day 16, *P* < 0.0001. (B) Oxygen consumption per protein mass is elevated in older animals. One-way ANOVA WT day 9 vs. day 12 vs. day 16, *P* < 0.001, one-way ANOVA *pnc-1* day 9 vs. day 12 vs. day 16, *P* < 0.0001. (C,D) DR reduces the overall respiration rate. One-way ANOVA WT AL vs. DR,*P* < 0.0001, two-way ANOVA WT AL/DR and age, *P* < 0.0003, one-way ANOVA *pnc-1*AL vs. DR,*P* < 0.032, two-way ANOVA *pnc-1*AL/DR and age, *P* < 0.0025. OCR is shown as normalized to worm number (C) and protein (D). Note that OCR values and trends are comparable in WT and *pnc-1(pk9605)* animals. AL data for WT and *pnc-1(pk9605)* that were used for normalization in (C,D) are shown in (A,B) and were obtained and analyzed in parallel to DR data. (E,F) DR increases the productive fraction of respiration, as detected by the increase in OCR seen upon administration of the mitochondrial uncoupler FCCP. Note that this trend was similar in WT and *pnc-1(pk9605)* animals. One-way ANOVA WT AL vs. DR,*P* < 0.023, two-way ANOVA WT AL vs. DR and age, *P* < 0.0635, one-way ANOVA *pnc-1*AL vs. DR,*P* < 0.011, two-way ANOVA *pnc-1*AL vs. DR and age, *P* < 0.0208, two-way ANOVA WT/*pnc-1* and age, *P* < 0.5548, two-way ANOVA WT/*pnc-1* and AL vs. DR,*P* < 0.0021. OCR is shown as normalized to worm number. Similar trends are seen in data normalized to protein content, presented in Fig. S3. In each experiment, samples were assayed in 3–5 replicates per experiment. Wells that did not respond were censored. Individual experimental data, mean, standard error, percent change, and statistical analysis are presented in (A) Table S23, S24, (B) Table S25, S26, (C) Table S27, (D) Table S28, (E) Table S23, S24, (F) Table S23, S24. *t-test vs. AL,*P* < 0.06; **t-test vs. AL,*P* < 0.01; @*t*-test vs. age 9, *P* < 0.055; #*t*-test vs. age 9, *P* < 0.01.

The evidence that DR promotes oxidative metabolism in yeast (Guarente, 2013; Schleit *et al*., 2013) predicts that this might also be true in *C. elegans*, even if DR reduces total oxygen consumption. This is an attractive model, because it seems logical that the efficiency of ATP production might need to be increased when food availability is reduced. To test this idea, we used the mitochondrial respiration uncoupler FCCP (Brand & Nicholls, 2011) to assess the extent to which oxygen consumption increases when it is uncoupled from ATP production. This increase reflects the proportion of respiratory capacity that was unused prior to uncoupling (Brand & Nicholls, 2011). The greater this increase, the more efficient the mitochondria were in generating ATP under basal conditions. At each day of life examined, the response to FCCP was dramatically higher in the DR group in either WT or *pnc-1* animals (Fig.5E,F, Tables S23 and S24). This suggests that under DR conditions, a greater proportion of oxygen consumption was devoted to ATP generation. Thus, although the overall respiration rate was not increased, DR induced a shift toward oxidative metabolism that might underlie the functions of NAD^+^-dependent mechanisms.

## Discussion

It has been proposed that DR lifespan extension is mediated through reduction of mTORC1 signaling, and activation of sirtuins and other NAD^+^-dependent mechanisms through an increase in respiration (Guarente, 2013; Johnson *et al*., 2013). Here, we used a new *C. elegans* liquid DR protocol to examine the importance of mechanisms that are associated with these two models. Our findings are consistent with some key predictions of each hypothesis, but suggest an important revision to the second model.

### Importance of stress defense and nutrient-sensing pathways in DR

We found that DR lifespan extension involved stress-defense regulators that are required in other longevity interventions (Kenyon, 2010), but were not necessarily essential in other *C. elegans* DR methods. One of these was DAF-16/FOXO (Figs2A and 6), which some but not all other studies implicated in DR (Greer *et al*., 2007; Greer & Brunet, 2009; Honjoh *et al*., 2009). Interestingly, the response to DR is improved by impairment of the insulin/IGF-1 receptor DAF-2, which inhibits DAF-16 (Bishop & Guarente, 2007). Together, these findings suggest that IIS, which senses nutrients, might be important in DR (Fig.6). Our DR lifespan extension also depended upon SKN-1/Nrf (Figs2C and 6), which is inhibited by IIS in parallel to DAF-16 (Tullet *et al*., 2008). Another study indicated that DR induces SKN-1 to increase respiration (Bishop & Guarente, 2007) but we observed that DR reduces oxygen consumption (Fig.5C,D), suggesting that SKN-1 has additional functions in DR. PHA-4/FOXA was also required for DR longevity in our protocol, although the background *smg-1(ts)* mutation substantially impaired the response to DR (Fig.2E). The SMG-1 kinase functions in nonsense-mediated decay, in which translationally stalled mRNAs are degraded (Sheaffer *et al*., 2008). Perhaps, clearing of stalled mRNAs by nonsense-mediated decay is important in DR.

**Figure 6:**
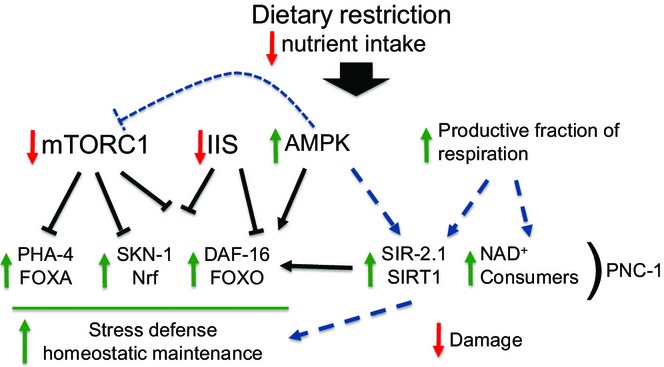
Importance of stress defense and metabolic mechanisms in DR. As described in the text, genetic experiments implicate the indicated growth-regulated stress-defense pathways and NAD^+^-associated mechanisms in DR. Other mechanisms that have been implicated in DR in *C. elegans* are consistent with this overall model. For example, both AMPK and PHA-4 promote autophagy (Hansen *et al*., 2008; Egan *et al*., 2011).

Each of these transcription factors is functionally inhibited by mTORC1 signaling (Fig.6) and required for longevity that results from inhibiting this pathway (Sheaffer *et al*., 2008; Robida-Stubbs *et al*., 2012). The proposed importance of mTORC1 in DR (Johnson *et al*., 2013) also fits with the importance of the low-energy sensor AMPK (Greer *et al*., 2007; Greer & Brunet, 2009) (Figs2G and 6), which inhibits mTORC1 in many species (Johnson *et al*., 2013). Our findings support the view that DR extends lifespan in part by strengthening stress defenses through a reduction in mTORC1 signaling, and possibly IIS (Fig.6). The importance of *daf-16*,*skn-1*, and *aak-2* that we observed differed from results of some *C. elegans* studies that examined these genes individually (see above). Perhaps, these mechanisms may control overlapping and possibly compensatory processes. It is also possible that our DR method has more stringent requirements for lifespan extension than some other protocols because the animals spend more time on AL feeding prior to DR (Table1).

### NAD^+^-dependent mechanisms, respiration, and DR

Our data also indicate the importance of NAD^+^-dependent mechanisms in DR and represent the first time that SIR-2.1/SIRT1 was required for DR lifespan extension in a *C. elegans* feeding protocol (Fig.3A,B). DR increased lifespan in *sir-2.1* mutants, which were slightly long-lived, but this lifespan extension was consistently reduced compared to WT across four experiments that were performed in our three laboratories (Table S8). The importance of SIR-2.1 in our DR regimen is consistent with the evidence that SIRT1 mediates many metabolic effects of DR in mammals (Haigis & Sinclair, 2010; Guarente, 2013), and strongly supports the idea that SIR-2.1/SIRT1 plays a major role in DR.

DR lifespan extension appeared to be less effective in *pnc-1* than *sir-2.1* mutants, revealing for the first time in a metazoan that the NAD^+^ salvage pathway is critical in DR, and suggesting that its importance might reflect the activity of NAD^+^ consumers besides SIR-2.1/SIRT1 (Fig.3A,G; Table2). However, DR improved two healthspan parameters independently of *pnc-1* (Fig.4; Tables S20 and S21), indicating that *pnc-1* mutants were not simply sick or completely refractory to DR. This result also indicates that some DR benefits may not require NAD^+^-mediated signals; therefore, that some effects of DR on healthspan can be uncoupled mechanistically from its longevity effects. Using *C. elegans* genetics, it may be possible to unravel how DR influences different parameters associated with health, and to elucidate how they contribute to long life.

**Table 2:** Maximum affect of DR on lifespan across mutant strains

Genotype	Average max % increase lifespan vs. AL	SEM	Food conc. (A_600_)	# Indep. expr.	*P-*value 2-way ANOVA vs. Ctrl.
N2	**85**	< 0.0	0.01	21	NA
*daf-16*	39	0.2	0.00	3	< 0.0001
*skn-1*	21	1.3	0.50	6	< 0.0001
*aak-2*	30	1.1	0.00	2	< 0.0001
*sir-2.1*	47	1.3	0.00	4	< 0.0001
*sir-2.1;sir-2.2;sir-2.4*	36	2.7	0.00	2	0.0001
*sir-2.1;sir-2.3;sir-2.4*	62	1.6	0.00	2	< 0.0001
*pnc-1*	32	0.9	0.50	10	< 0.0001
*smg-1*	**45**	2.5	0.00	4	NA
*pha-4;smg-1*	10	1.0	0.00	4	0.0004

This table presents the DR food concentration at which each strain experiences its maximal lifespan extension, in comparison to its lifespan on AL food, as well as what the percent increase in lifespan is. The wild-type N2 is the control for all strains except for *pha-4;smg-1,* whose genetic control is *smg-1*.

The percent change experienced by the control strains is in bold.

It is an important question which NAD^+^ consumers besides SIR-2.1/SIRT1 might contribute to DR lifespan extension. Poly-ADP ribose polymerase (PARP) proteins consume NAD^+^ but are unlikely to play a positive role in DR because reducing their activity increases *C. elegans* lifespan (Mouchiroud *et al*., 2013a). However, we cannot exclude an important function for the predicted redundant sirtuins SIR-2.2 and SIR-2.3 (SIRT4; Fig.3C,E), and in some tissues, NAD^+^ generated by PNC-1 might maintain proper levels of NADP (and NADPH) or activity of the many NAD^+^-dependent metabolic processes (Pollak *et al*., 2007). It might also be critical for PNC-1 to metabolize NAM, excess levels of which recapitulate some developmental *pnc-1* phenotypes (Vrablik *et al*., 2009). Administration of exogenous NAM extends *C. elegans* lifespan by means of metabolites that increase ROS formation (Schmeisser *et al*., 2013), but a positive role for NAM in DR seems unlikely given that DR requires PNC-1, which metabolizes NAM.

Our surprising finding that DR sharply reduced the overall OCR (Fig.5C,D; Tables S23–S28) would seem to be strong evidence against the view that DR exerts its beneficial effects by increasing respiration (Bishop & Guarente, 2007; Guarente, 2013). Importantly, however, DR increased the proportion of oxygen consumption that is devoted to ATP generation (Fig.5E; Tables S23 and S24). This shift toward oxidative metabolism, which we documented for the first time in a metazoan, seems to be a fitting response to lower food availability because respiration is more efficient than glycolysis. It will be of interest to determine whether this shift is also seen in other *C. elegans* DR protocols, including those involving solid culture media. Our data suggest a revised version of the respiration-based model for DR, whereby the driving force for activity of NAD^+^-dependent processes that delay aging is this respiratory shift (Fig.6), not increased respiration *per se*. This respiratory shift might also influence the mitochondrial unfolded response and possibly other signals from mitochondria that could affect lifespan (Houtkooper *et al*., 2013; Mouchiroud *et al*., 2013b; Schleit *et al*., 2013; Schmeisser *et al*., 2013). The next challenge will be to test these ideas by identifying mechanisms that are modulated by SIR-2.1 and other key NAD^+^-dependent processes in the setting of DR, and elucidating how they and other mitochondrial-associated processes are affected by the metabolic changes that are driven by DR.

## Experimental procedures

Full methods and experimental procedures are available in Data S1 (Supporting Information).

## Supporting Information

**Fig. S1** The NAD^+^ salvage pathway. **Fig. S2** Analysis of treated bacteria. **Fig. S3** Effects of the Mitochondrial Uncoupler FCCP on AL and DR animals. **Fig. S4** Regulation of longevity transcription factors by the IIS and TORC1 pathways.

Table S1 Comparison of published *C. elegans* liquid DR methods.

Table S2 Bacterial concentration conversion table.

**Table S3** Statistical analysis of compiled data of wild-type animals on DR shown in Fig. 1B,C. **Table S4** Statistical analysis of compiled data of *daf-16* mutant animals on DR shown in Fig. 2A,B. **Table S5** Statistical analysis of compiled data of *skn-1* mutant animals on DR shown in Fig. 2C,D. **Table S6** Statistical analysis of compiled data of *pha-4;smg-1* mutant animals on DR shown in Fig. 2E,F. **Table S7** Statistical analysis of compiled data of *aak-2* mutant animals on DR shown in Fig. 2G,H. **Table S8** Statistical analysis of compiled data of *sir-2.1* mutant animals on DR shown in in Fig. 3A,B. **Table S9** Statistical analysis of compiled data of *sir-2.1;sir-2.2;sir-2.4* mutant animals on DR shown in Fig. 3C,D. **Table S10** Statistical analysis of compiled data of *sir-2.1;sir-2.3;sir-2.4* mutant animals on DR shown in Fig. 3E,F. **Table S11** Statistical analysis of compiled data of *pnc-1* mutant animals on DR shown in Fig. 3G,H.

Table S12 Individual DR experiments performed on wild-type animals shown in Fig. 1B,C.

Table S13 Individual DR experiments performed on *daf-16* animals shown in Fig. 2A,B.

Table S14 Individual DR experiments performed on *skn-1* animals shown in Fig. 2C,D.

Table S15 Individual DR experiments performed on *pha-4;smg-1* animals shown in Fig. 2E,F.

Table S16 Individual DR experiments performed on *aak-2* animals shown in Fig. 2G,H.

Table S17 Individual DR experiments performed on *sir-2.1* animals shown in Fig. 3A,B.

Table S18 Individual DR experiments performed on *sir-2.1;sir-2.2;sir-2.4* animals shown in Fig. 3C,D.

Table S19 Individual DR experiments performed on *sir-2.1;sir-2.3;sir-2.4* animals shown in Fig. 3E,F.

Table S20 Individual DR experiments performed on *pnc-1* animals shown in Fig. 3G,H.

Table S21 Analyses of thrashing assays shown in Fig. 4A,B.

Table S22 Analyses of thermotolerance assays shown in Fig. 4C,D.

Table S23 Respiration under basal conditions per animal shown in Fig. 5A,E,F.

Table S24 Uncoupled respiration data per animal shown in Fig. 5E,F.

Table S25 Respiration under basal conditions per protein shown in Fig. 5B, Fig. S3A,B.

Table S26 Uncoupled respiration per protein shown in Fig. S3A,S3B.

Table S27 Respiration per animal normalized to ad-lib conditions shown in Fig. 5C.

Table S28 Respiration per protein normalized to ad-lib conditions shown in Fig. 5D.

Data S1 Experimental procedures.
