# Supplementary material for: Dietary restriction involves NAD+-dependent mechanisms and a shift toward oxidative metabolism
Source: Aging Cell. 2014 Sep 25;13(6):1075–85. doi: 10.1111/acel.12273 (PMC4244309; doi:10.1111/acel.12273)
Supplement: Supplementary file 1 [file acel0013-1075-sd10.docx]

| Supplemental Table 17. Analyses of individual DR experiments performed on *sir-2.1* animals shown in Figure 3A and 3B. | | | | | | | | | | | | | |
| --- | --- | --- | --- | --- | --- | --- | --- | --- | --- | --- | --- | --- | --- |
|  |  |  |  |  |  |  |  |  |  |  |  |  |  |
| **Strain** | **OD600** | **Exp Mean Lifespan (days)** | **Normalized Exp Mean Lifespan (days)** | **Exp 75th Percentile (days)** | **# Exp Animals** | **% increase lifespan vs AL** | **N2 Mean Lifespan (days)** | **Normalized N2 Mean Lifespan (days)** | **N2 75th Percentile (days)** | **# N2 animals** | **N2 % lifespan increase vs AL** | **Exp vs N2 % increase lifespan** | **ID** |
| *sir-2.1* | 3.00 | 29.1 | 1.00 | 35 | 67 | 0.0 | 27.0 | 1.00 | 32 | 64 | 7.9 | 0.0 | 7a* |
|  | 1.50 | 39.0 | 1.34 | 45 | 60 | 34.1 | 37.5 | 1.39 | 42 | 72 | 3.9 | 39.3 | 7a* |
|  | 0.50 | 38.9 | 1.34 | 40 | 47 | 33.8 | 41.4 | 1.54 | 45 | 63 | -6.1 | 53.6 | 7a* |
|  | 0.30 | 35.8 | 1.23 | 38 | 64 | 23.0 | 39.7 | 1.47 | 42 | 66 | -10.0 | 47.3 | 7a* |
|  | 0.15 | 35.5 | 1.22 | 38 | 84 | 22.1 | 39.3 | 1.46 | 42 | 59 | -9.7 | 46.0 | 7a* |
|  | 0.05 | 35.5 | 1.22 | 38 | 68 | 22.3 | 40.1 | 1.49 | 45 | 86 | -11.3 | 48.7 | 7a* |
|  | 0.01 | 39.6 | 1.36 | 42 | 60 | 36.3 | 47.1 | 1.75 | 55 | 78 | -15.9 | 74.9 | 7a* |
|  | 0.00 | 47.4 | 1.63 | 55 | 66 | 63.1 | 52.1 | 1.93 | 57 | 77 | -9.0 | 93.4 | 7a* |
|  |  |  |  |  |  |  |  |  |  |  |  |  |  |
|  | 3.00 | 27.2 | 1.00 | 31 | 50 | 0.0 | 24.0 | 1.00 | 31 | 55 | 13.6 | 0.0 | 7b* |
|  | 1.50 | 32.1 | 1.18 | 38 | 43 | 17.9 | 31.5 | 1.32 | 40 | 61 | 1.8 | 31.6 | 7b* |
|  | 0.50 | 38.1 | 1.40 | 42 | 48 | 40.1 | 38.5 | 1.61 | 46 | 57 | -1.1 | 60.8 | 7b* |
|  | 0.30 | 35.6 | 1.31 | 40 | 52 | 30.8 | 36.8 | 1.53 | 40 | 63 | -3.2 | 53.5 | 7b* |
|  | 0.15 | 34.2 | 1.26 | 40 | 55 | 25.6 | 35.9 | 1.50 | 40 | 56 | -4.7 | 49.7 | 7b* |
|  | 0.05 | 30.6 | 1.12 | 38 | 43 | 12.4 | 40.3 | 1.68 | 46 | 53 | -24.1 | 68.3 | 7b* |
|  | 0.01 | 30.0 | 1.10 | 35 | 45 | 10.4 | 43.1 | 1.80 | 53 | 55 | -30.3 | 80.0 | 7b* |
|  | 0.00 | 31.6 | 1.16 | 38 | 49 | 16.1 | 46.4 | 1.93 | 53 | 56 | -31.9 | 93.4 | 7b* |
|  |  |  |  |  |  |  |  |  |  |  |  |  |  |
|  | 3.00 | 26.1 | 1.00 | 29 | 62 | 0.0 | 22.5 | 1.00 | 24 | 13 | 16.4 | 0.0 | 7c |
|  | 1.50 | 38.0 | 1.45 | 45 | 154 | 45.2 | 22.1 | 0.99 | 23 | 7 | 71.5 | -1.4 | 7c |
|  | 0.50 | 39.6 | 1.51 | 46 | 116 | 51.4 | 39.5 | 1.76 | 46 | 10 | 0.2 | 75.9 | 7c |
|  | 0.30 | 36.4 | 1.39 | 38 | 53 | 39.1 | NA | NA | NA | NA | NA | NA | 7c |
|  | 0.15 | 33.6 | 1.28 | 34 | 153 | 28.3 | 43.6 | 1.94 | 45 | 9 | -23.0 | 94.0 | 7c |
|  | 0.01 | 35.3 | 1.35 | 38 | 97 | 34.9 | 37.8 | 1.68 | 41 | 10 | -6.7 | 68.4 | 7c |
|  | 0.00 | 41.9 | 1.60 | 46 | 83 | 60.1 | 44.1 | 1.97 | 51 | 22 | -5.1 | 96.5 | 7c |
|  |  |  |  |  |  |  |  |  |  |  |  |  |  |
|  | 3.00 | 27.1 | 1.00 | 30 | 83 | 0.0 | 22.3 | 1.00 | 23 | 31 | 21.7 | 0.0 | 7d |
|  | 1.50 | 26.7 | 0.98 | 30 | 100 | -1.5 | 23.2 | 1.04 | 25 | 63 | 15.2 | 4.0 | 7d |
|  | 0.50 | 37.0 | 1.37 | 40 | 85 | 36.6 | 41.8 | 1.87 | 44 | 36 | -11.3 | 87.4 | 7d |
|  | 0.30 | 39.3 | 1.45 | 44 | 131 | 45.1 | 36.9 | 1.65 | 44 | 16 | 6.7 | 65.4 | 7d |
|  | 0.15 | 38.6 | 1.42 | 44 | 74 | 42.1 | 43.9 | 1.97 | 44 | 14 | -12.2 | 97.1 | 7d |
|  | 0.01 | 36.4 | 1.34 | 39 | 86 | 34.1 | 43.9 | 1.97 | 44 | 17 | -17.1 | 96.9 | 7d |
|  | 0.00 | 40.8 | 1.50 | 44 | 76 | 50.4 | 38.8 | 1.74 | 44 | 25 | 5.0 | 74.2 | 7d |
| ID* indicates experiments performed in the Sinclair/Hart lab. All others were performed in the Blackwell Lab. | | | | | | | | |  |  |  |  |  |

Experimental animals refer to *sir-2.1* mutants. Data are organized and analyzed as in Table S12.
