# Supplementary material for: Dietary restriction involves NAD+-dependent mechanisms and a shift toward oxidative metabolism
Source: Aging Cell. 2014 Sep 25;13(6):1075–85. doi: 10.1111/acel.12273 (PMC4244309; doi:10.1111/acel.12273)
Supplement: Supplementary file 2 [file acel0013-1075-sd11.docx]

| Supplemental Table 18. Analyses of individual DR experiments performed on *sir-2.1;sir-2.2;sir-2.4* animals shown in Figure 3C and 3D. | | | | | | | | | | | | | |
| --- | --- | --- | --- | --- | --- | --- | --- | --- | --- | --- | --- | --- | --- |
|  |  |  |  |  |  |  |  |  |  |  |  |  |  |
| **Strain** | **OD600** | **Exp Mean Lifespan (days)** | **Normalized Exp Mean Lifespan (days)** | **Exp 75th Percentile (days)** | **# Exp Animals** | **% increase lifespan vs AL** | **N2 Mean Lifespan (days)** | **Normalized N2 Mean Lifespan (days)** | **N2 75th Percentile (days)** | **# N2 animals** | **N2 % lifespan increase vs AL** | **Exp vs N2 % increase lifespan** | **ID** |
| *sir-2.1;sir-2.2;sir-2.4* | 3.00 | 24.7 | 1.00 | 27 | 60 | 0.0 | 21.7 | 1.00 | 25 | 35 | 0.0 | 13.7 | 8a |
|  | 0.50 | 29.6 | 1.20 | 38 | 78 | 20.0 | 41.2 | 1.90 | 48 | 28 | 90.0 | -28.2 | 8a |
|  | 0.25 | 28.7 | 1.16 | 38 | 35 | 16.2 | 39.3 | 1.81 | 46 | 42 | 81.1 | -27.1 | 8a |
|  | 0.05 | 26.7 | 1.08 | 32 | 27 | 8.1 | 40.5 | 1.87 | 42 | 28 | 86.9 | -34.2 | 8a |
|  | 0.00 | 29.8 | 1.21 | 34 | 29 | 20.6 | 37.5 | 1.73 | 42 | 33 | 72.7 | -20.5 | 8a |
|  |  |  |  |  |  |  |  |  |  |  |  |  |  |
|  | 3.00 | 28.7 | 1.00 | 31 | 37 | 0.0 | 24.9 | 1.00 | 29 | 31 | 0.0 | 15.3 | 8b |
|  | 0.50 | 31.4 | 1.09 | 36 | 44 | 9.4 | 36.1 | 1.45 | 44 | 34 | 45.1 | -13.0 | 8b |
|  | 0.25 | 38.5 | 1.34 | 50 | 45 | 34.3 | 40.7 | 1.64 | 50 | 30 | 63.6 | -5.4 | 8b |
|  | 0.05 | 36.7 | 1.28 | 44 | 26 | 27.8 | 43.1 | 1.73 | 50 | 34 | 73.1 | -14.9 | 8b |
|  | 0.00 | 43.6 | 1.52 | 51 | 39 | 52.2 | 43.4 | 1.74 | 50 | 37 | 74.3 | 0.7 | 8b |
|  |  |  |  |  |  |  |  |  |  |  |  |  |  |
|  |  |  |  |  |  |  |  |  |  |  |  |  |  |

Experimental animals refer to *sir-2.1;sir-2.2;sir-2.4* mutants. Data are organized and analyzed as in Table S12.
