# Supplementary material for: Dietary restriction involves NAD+-dependent mechanisms and a shift toward oxidative metabolism
Source: Aging Cell. 2014 Sep 25;13(6):1075–85. doi: 10.1111/acel.12273 (PMC4244309; doi:10.1111/acel.12273)
Supplement: Supplementary file 3 [file acel0013-1075-sd12.docx]

| Supplemental Table 19. Analyses of individual DR experiments performed on *sir-2.1;sir-2.3;sir-2.4* animals shown in Figure 3E and 3F. | | | | | | | | | | | | | |
| --- | --- | --- | --- | --- | --- | --- | --- | --- | --- | --- | --- | --- | --- |
|  |  |  |  |  |  |  |  |  |  |  |  |  |  |
| **Strain** | **OD600** | **Exp Mean Lifespan (days)** | **Normalized Exp Mean Lifespan (days)** | **Exp 75th Percentile (days)** | **# Exp Animals** | **% increase lifespan vs AL** | **N2 Mean Lifespan (days)** | **Normalized N2 Mean Lifespan (days)** | **N2 75th Percentile (days)** | **# N2 animals** | **N2 % lifespan increase vs AL** | **Exp vs N2 % increase lifespan** | **ID** |
| *sir-2.1;sir-2.3;sir-2.4* | 3.00 | 24.6 | 1.00 | 27 | 190 | 0.0 | 22.3 | 1.00 | 23 | 31 | 0.0 | 10.1 | 9a |
|  | 1.50 | 27.1 | 1.10 | 30 | 271 | 10.3 | 23.2 | 1.04 | 25 | 63 | 4.0 | 16.9 | 9a |
|  | 0.50 | 37.0 | 1.51 | 39 | 108 | 50.9 | 41.8 | 1.87 | 44 | 36 | 87.4 | -11.3 | 9a |
|  | 0.30 | 35.8 | 1.46 | 38 | 420 | 45.7 | 36.9 | 1.65 | 44 | 16 | 65.4 | -3.0 | 9a |
|  | 0.05 | 36.7 | 1.49 | 38 | 317 | 49.3 | 43.9 | 1.97 | 44 | 17 | 96.9 | -16.5 | 9a |
|  | 0.00 | 43.5 | 1.77 | 44 | 153 | 77.1 | 38.8 | 1.74 | 44 | 25 | 74.2 | 11.9 | 9a |
|  |  |  |  |  |  |  |  |  |  |  |  |  |  |
|  | 3.00 | 22.6 | 1.00 | 25 | 100 | 0.0 | 21.7 | 1.00 | 25 | 35 | 0.0 | 4.1 | 9b |
|  | 0.50 | 35.2 | 1.56 | 44 | 48 | 55.6 | 41.2 | 1.90 | 48 | 28 | 90.0 | -14.7 | 9b |
|  | 0.30 | 35.8 | 1.58 | 47 | 46 | 58.4 | 39.3 | 1.81 | 46 | 42 | 81.1 | -8.9 | 9b |
|  | 0.05 | 34.6 | 1.53 | 44 | 51 | 53.3 | 40.5 | 1.87 | 42 | 28 | 86.9 | -14.6 | 9b |
|  | 0.00 | 33.1 | 1.47 | 39 | 36 | 46.6 | 37.5 | 1.73 | 42 | 33 | 72.7 | -11.6 | 9b |

Experimental animals refer to *sir-2.1;sir-2.3;sir-2.4* mutants. Data are organized and analyzed as in Table S12.
