# Supplementary material for: Dietary restriction involves NAD+-dependent mechanisms and a shift toward oxidative metabolism
Source: Aging Cell. 2014 Sep 25;13(6):1075–85. doi: 10.1111/acel.12273 (PMC4244309; doi:10.1111/acel.12273)
Supplement: Supplementary file 4 [file acel0013-1075-sd13.docx]

| Supplemental Table 20. Analyses of individual DR experiments performed on *pnc-1* animals shown in Figure 3G and 3H. | | | | | | | | | | | | | |
| --- | --- | --- | --- | --- | --- | --- | --- | --- | --- | --- | --- | --- | --- |
|  |  |  |  |  |  |  |  |  |  |  |  |  |  |
| **Strain** | **OD600** | **Exp Mean Lifespan (days)** | **Normalized Exp Mean Lifespan (days)** | **Exp 75th Percentile (days)** | **# Exp Animals** | **% increase lifespan vs AL** | **N2 Mean Lifespan (days)** | **Normalized N2 Mean Lifespan (days)** | **N2 75th Percentile (days)** | **# N2 animals** | **N2 % lifespan increase vs AL** | **Exp vs N2 % increase lifespan** | **ID** |
| *pnc-1* | 3.00 | 21.2 | 1.00 | 28 | 57 | 0.0 | 28.8 | 1.00 | 33 | 57 | 0.0 | -26.2 | 3a* |
|  | 1.50 | 26.6 | 1.25 | 33 | 60 | 25.2 | 38.7 | 1.34 | 47 | 50 | 34.4 | -31.2 | 3a* |
|  | 0.50 | 27.7 | 1.30 | 38 | 56 | 30.2 | 46.1 | 1.60 | 52 | 57 | 60.2 | -40.0 | 3a* |
|  | 0.30 | 24.9 | 1.17 | 33 | 47 | 17.1 | 47.0 | 1.63 | 52 | 61 | 63.5 | -47.1 | 3a* |
|  | 0.15 | 20.3 | 0.96 | 28 | 32 | -4.2 | 41.4 | 1.44 | 47 | 45 | 43.9 | -50.9 | 3a* |
|  | 0.05 | 21.0 | 0.99 | 28 | 25 | -1.2 | 42.6 | 1.48 | 45 | 52 | 48.1 | -50.7 | 3a* |
|  | 0.05 | 20.2 | 0.95 | 31 | 26 | -4.8 | 48.9 | 1.70 | 54 | 52 | 70.0 | -58.6 | 3a* |
|  |  |  |  |  |  |  |  |  |  |  |  |  |  |
|  | 3.00 | 21.2 | 1.00 | 20 | 39 | 0.0 | 21.6 | 1.00 | 24 | 18 | 0.0 | -2.0 | 3b |
|  | 1.50 | 22.0 | 1.04 | 24 | 52 | 4.1 | 24.6 | 1.14 | 23 | 27 | 13.8 | -10.4 | 3b |
|  | 0.50 | 26.8 | 1.26 | 31 | 57 | 26.5 | 37.1 | 1.72 | 44 | 17 | 71.7 | -27.8 | 3b |
|  | 0.30 | 28.9 | 1.37 | 34 | 73 | 36.8 | 41.2 | 1.91 | 45 | 40 | 90.9 | -29.8 | 3b |
|  | 0.15 | 24.8 | 1.17 | 31 | 44 | 17.4 | 36.3 | 1.68 | 40 | 22 | 68.2 | -31.6 | 3b |
|  | 0.05 | 31.4 | 1.48 | 37 | 74 | 48.5 | 43.4 | 2.01 | 45 | 36 | 100.9 | -27.6 | 3b |
|  | 0.00 | 30.0 | 1.42 | 37 | 130 | 41.8 | 44.0 | 2.04 | 52 | 29 | 103.8 | -31.8 | 3b |
|  |  |  |  |  |  |  |  |  |  |  |  |  |  |
|  | 3.00 | 19.5 | 1.00 | 21 | 28 | 0.0 | 19.7 | 1.00 | 21 | 29 | 0.0 | -1.1 | 3c |
|  | 0.50 | 22.1 | 1.14 | 26 | 20 | 13.6 | 34.0 | 1.73 | 41 | 34 | 72.5 | -34.9 | 3c |
|  | 0.00 | 22.6 | 1.16 | 28 | 41 | 15.9 | 33.4 | 1.70 | 41 | 40 | 69.6 | -32.5 | 3c |
|  |  |  |  |  |  |  |  |  |  |  |  |  |  |
|  | 3.00 | 21.9 | 1.00 | 26 | 59 | 0.0 | 21.7 | 1.00 | 25 | 35 | 0.0 | 1.1 | 3d |
|  | 0.50 | 28.0 | 1.28 | 33 | 33 | 27.9 | 41.2 | 1.90 | 48 | 28 | 90.0 | -32.0 | 3d |
|  | 0.30 | 25.2 | 1.15 | 36 | 26 | 14.9 | 39.3 | 1.81 | 46 | 42 | 81.1 | -35.9 | 3d |
|  | 0.05 | 25.6 | 1.17 | 36 | 23 | 16.7 | 40.5 | 1.87 | 42 | 28 | 86.9 | -36.9 | 3d |
|  | 0.00 | 19.2 | 0.87 | 30 | 26 | -12.6 | 37.5 | 1.73 | 42 | 33 | 72.7 | -48.9 | 3d |
|  |  |  |  |  |  |  |  |  |  |  |  |  |  |
|  | 3.00 | 20.8 | 1.00 | 22 | 25 | 0.0 | 25.2 | 1.00 | 28 | 30 | 0.0 | -17.4 | 3e |
|  | 3.00 | 21.7 | 1.00 | 25 | 35 | 0.0 | NA | 1.00 | NA | NA | NA | -13.9 | 3e |
|  | 3.00 | 21.7 | 1.00 | 25 | 29 | 0.0 | NA | 1.00 | NA | NA | NA | -13.8 | 3e |
|  | 0.50 | 33.8 | 1.63 | 44 | 28 | 62.5 | 42.7 | 1.69 | 46 | 56 | 69.3 | -20.8 | 3e |
|  | 0.50 | 31.8 | 1.46 | 39 | 34 | 46.5 | NA | 1.69 | NA | NA | NA | -25.5 | 3e |
|  | 0.50 | 31.7 | 1.46 | 39 | 40 | 46.0 | NA | 1.69 | NA | NA | NA | -25.6 | 3e |
|  |  |  |  |  |  |  |  |  |  |  |  |  |  |
|  | 3.00 | 23.4 | 1.00 | 29 | 24 | 0.0 | 29.8 | 1.00 | 35 | 36 | 0.0 | -21.4 | 3f |
|  | 3.00 | NA | NA | NA | NA | NA | 26.0 | 1.00 | 33 | 32 | 0.0 | NA | 3f |
|  | 3.00 | NA | NA | NA | NA | NA | 28.3 | 1.00 | 35 | 41 | 0.0 | NA | 3f |
|  | 0.50 | 25.5 | 1.09 | 32 | 16 | 8.9 | 36.1 | 1.21 | 40 | 33 | 21.0 | -29.3 | 3f |
|  | 0.50 | 28.2 | 1.20 | 39 | 16 | 20.4 | 37.1 | 1.43 | 43 | 38 | 42.8 | -24.0 | 3f |
|  | 0.50 | NA | NA | NA | NA | NA | 34.3 | 1.21 | 42 | 36 | 21.3 | NA | 3f |
|  |  |  |  |  |  |  |  |  |  |  |  |  |  |
|  | 3.00 | 21.8 | 1.00 | 28 | 36 | 0.0 | 25.9 | 1.00 | 28 | 33 | 0.0 | -16.0 | 3g |
|  | 3.00 | NA | NA | NA | NA | NA | 25.9 | 1.00 | 28 | 41 | 0.0 | NA | 3g |
|  | 0.50 | 30.2 | 1.39 | 37 | 31 | 38.6 | 38.8 | 1.50 | 43 | 41 | 49.8 | -22.3 | 3g |
|  | 0.50 | NA | NA | NA | NA | NA | 38.7 | 1.49 | 47 | 35 | 49.2 | NA | 3g |
| ID* indicates experiments performed in the Sinclair/Hart lab. All others were performed in the Blackwell Lab. | | | | | | | | |  |  |  |  |  |

Experimental animals refer to *pnc-1* mutants. Data are organized and analyzed as in Table S12.
