# Supplementary material for: Dietary restriction involves NAD+-dependent mechanisms and a shift toward oxidative metabolism
Source: Aging Cell. 2014 Sep 25;13(6):1075–85. doi: 10.1111/acel.12273 (PMC4244309; doi:10.1111/acel.12273)
Supplement: Supplementary file 5 [file acel0013-1075-sd14.docx]

| Supplemental Table 21. Analyses of individual thrashing assays shown in Figure 4A and 4B. | | | | | | | | | | | | |
| --- | --- | --- | --- | --- | --- | --- | --- | --- | --- | --- | --- | --- |
|  |  |  |  |  |  |  |  |  |  |  |  |  |
| **strain** | **age (days)** | **condition** | **mean # thrashes (min^-1^)** | **SEM** | **N** | **avg thrashing (min^-1^)** | **SEM** | **% increase vs. AL** | **avg % increase vs. AL** | **SEM** | **% increase vs day 10** | **exp #** |
| N2 | 13 | AL | 21.0 | 1.7 | 10 | 36.1 | 1.2 |  |  |  |  | 1 |
|  |  |  | 42.1 | 3.1 | 10 |  |  |  |  |  |  | 2 |
|  |  |  | 45.1 | 4.5 | 10 |  |  |  |  |  |  | 3 |
|  |  | DR | 55.4 | 3.5 | 10 | 90.6 | 3.9 | 164.2 | 152.2 | 11.2 |  | 1 |
|  |  |  | 78.1 | 3.0 | 10 |  |  | 85.7 |  |  |  | 2 |
|  |  |  | 138.4 | 4.5 | 10 |  |  | 206.6 |  |  |  | 3 |
| *pnc-1* | 13 | AL | 21.8 | 2.8 | 10 | 22.6 | 0.1 |  |  |  |  | 1 |
|  |  |  | 23.7 | 1.5 | 10 |  |  |  |  |  |  | 2 |
|  |  |  | 22.4 | 1.3 | 10 |  |  |  |  |  |  | 3 |
|  |  | DR | 40.4 | 2.2 | 10 | 58.1 | 1.4 | 85.3 | 155.8 | 11.9 |  | 1 |
|  |  |  | 63.5 | 3.5 | 10 |  |  | 168.3 |  |  |  | 2 |
|  |  |  | 70.3 | 2.8 | 10 |  |  | 213.7 |  |  |  | 3 |
|  |  |  |  |  |  |  |  |  |  |  |  |  |
| N2 | 16 | AL | 27.5 | 1.9 | 10 | 23.5 | 0.3 |  |  |  | -35.5 | 4 |
|  |  |  | 22.9 | 1.5 | 10 |  |  |  |  |  |  | 5 |
|  |  |  | 20.1 | 1.9 | 10 |  |  |  |  |  |  | 6 |
|  |  | DR | 75.1 | 3.2 | 10 | 67.8 | 0.7 | 172.8 | 190.0 | 2.7 | 24.9 | 4 |
|  |  |  | 68.3 | 2.8 | 10 |  |  | 198.4 |  |  |  | 5 |
|  |  |  | 60.1 | 3.3 | 10 |  |  | 198.7 |  |  |  | 6 |
| *pnc-1* | 16 | AL | 15.9 | 0.9 | 10 | 16.6 | 0.1 |  |  |  | -28.0 | 4 |
|  |  |  | 17.9 | 1.1 | 10 |  |  |  |  |  |  | 5 |
|  |  |  | 16.0 | 1.9 | 10 |  |  |  |  |  |  | 6 |
|  |  | DR | 48.8 | 3.1 | 10 | 45.5 | 0.3 | 207.6 | 175.8 | 5.8 | 36.2 | 4 |
|  |  |  | 43.8 | 2.7 | 10 |  |  | 144.0 |  |  |  | 5 |
|  |  |  | 44.0 | 1.5 | 10 |  |  | 175.8 |  |  |  | 6 |

Individual body movement/thrashing assays were performed on WT and *pnc-1* mutant animals. Experiments with different numbers were performed independently of one another. AL refers to a bacterial OD600 of 3 and DR, a bacterial OD600 of 0.5. Percentage increase in thrashing = (mean thrashing at DR - mean thrashing at AL performed in parallel)/mean thrashing at AL performed in parallel x 100. N represents the number of animals analyzed. Further description can be found in the methods section.
