# Supplementary material for: Dietary restriction involves NAD+-dependent mechanisms and a shift toward oxidative metabolism
Source: Aging Cell. 2014 Sep 25;13(6):1075–85. doi: 10.1111/acel.12273 (PMC4244309; doi:10.1111/acel.12273)
Supplement: Supplementary file 6 [file acel0013-1075-sd15.docx]

| Supplemental Table 22. Analyses of individual thermotolerance assays shown in Figure 4C and 4D. | | | | | | | | | | | | |
| --- | --- | --- | --- | --- | --- | --- | --- | --- | --- | --- | --- | --- |
| **strain** | **age (days)** | **condition** | **mean survival** | **SE** | **N** | **avg** | **SE** | **% increase vs. AL** | **avg % increase vs. AL** | **SE** | **% increase vs day 10** | **exp #** |
| N2 | 10 | AL | 20.3 | 0.7 | 27 | 19.5 | 0.1 |  |  |  |  | 1 |
|  |  |  | 20.3 | 2.9 | 31 |  |  |  |  |  |  | 2 |
|  |  |  | 18.0 | 2.2 | 48 |  |  |  |  |  |  | 3 |
|  |  | DR | 19.3 | 1.8 | 30 | 17.5 | 0.2 | -4.8 | -10.3 | 1.1 |  | 1 |
|  |  |  | 15.2 | 0.9 | 62 |  |  | -25.1 |  |  |  | 2 |
|  |  |  | 17.9 | 2.0 | 52 |  |  | -0.9 |  |  |  | 3 |
| *pnc-1* | 10 | AL | 16.9 | 3.1 | 15 | 15.3 | 0.2 |  |  |  |  | 1 |
|  |  |  | 13.6 | 0.2 | 56 |  |  |  |  |  |  | 2 |
|  |  |  | 15.3 | 0.4 | 45 |  |  |  |  |  |  | 3 |
|  |  | DR | 13.5 | 0.2 | 19 | 14.6 | 0.1 | -20.0 | -3.1 | 1.8 |  | 1 |
|  |  |  | 15.9 | 2.4 | 54 |  |  | 17.0 |  |  |  | 2 |
|  |  |  | 14.4 | 1.1 | 36 |  |  | -6.4 |  |  |  | 3 |
|  |  |  |  |  |  |  |  |  |  |  |  |  |
| N2 | 15 | AL | 9.3 | 0.3 | 46 | 12.7 | 0.3 |  |  |  | -35.1 | 4 |
|  |  |  | 11.1 | 1.0 | 55 |  |  |  |  |  |  | 5 |
|  |  |  | 17.6 | 1.0 | 63 |  |  |  |  |  |  | 6 |
|  |  | DR | 19.8 | 5.2 | 31 | 21.8 | 0.2 | 112.3 | 81.4 | 3.5 | 24.9 | 4 |
|  |  |  | 21.7 | 4.1 | 49 |  |  | 96.3 |  |  |  | 5 |
|  |  |  | 23.9 | 0.4 | 57 |  |  | 35.5 |  |  |  | 6 |
| *pnc-1* | 15 | AL | 10.8 | 1.0 | 20 | 11.0 | 0.1 |  |  |  | -28.0 | 4 |
|  |  |  | 10.3 | 0.6 | 53 |  |  |  |  |  |  | 5 |
|  |  |  | 12.0 | 2.8 | 61 |  |  |  |  |  |  | 6 |
|  |  | DR | 15.8 |  | 12 | 19.9 | 0.5 | 47.3 | 79.7 | 3.0 | 36.2 | 4 |
|  |  |  | 19.1 | 3.1 | 24 |  |  | 86.0 |  |  |  | 5 |
|  |  |  | 24.8 | 2.4 | 61 |  |  | 105.9 |  |  |  | 6 |

Individual 38°C heat stress assays were performed on WT and *pnc-1* mutant animals. Experiments with different numbers were performed independently of one another. AL refers to a bacterial OD600 of 3 and DR, a bacterial OD600 of 0.5. Percentage increase in survival = (mean survival at DR - mean survival at AL performed in parallel)/mean survival at AL performed in parallel x 100. N represents the number of animals analyzed. Further description can be found in the methods section.
