# Supplementary material for: Dietary restriction involves NAD+-dependent mechanisms and a shift toward oxidative metabolism
Source: Aging Cell. 2014 Sep 25;13(6):1075–85. doi: 10.1111/acel.12273 (PMC4244309; doi:10.1111/acel.12273)
Supplement: Supplementary file 7 [file acel0013-1075-sd16.docx]

| Supplemental Table 23. Respiration under basal conditions analyzed per animal shown in Figures 5A, 5E, and 5F. | | | | | | | | | | | | | | |
| --- | --- | --- | --- | --- | --- | --- | --- | --- | --- | --- | --- | --- | --- | --- |
|  |  |  |  |  |  |  |  |  |  |  |  |  |  |  |
| **strain** | **age (days)** | **condition** | **mean OCR/N** | **SEM** | **avg** | **SEM** | **% increase vs. AL** | **avg % increase vs. AL** | **SEM** | **avg % increase vs day 9** | ***p* value vs N2** | ***p value* vs AL** | ***p value* vs day 9** | **exp #** |
| N2 | 9 | AL | 10.71 | 0.76 | 11.06 | 0.25 |  |  |  |  |  |  |  | 1 |
|  |  |  | 10.92 | 1.21 |  |  |  |  |  |  |  |  |  | 2 |
|  |  |  | 11.55 | 0.60 |  |  |  |  |  |  |  |  |  | 4 |
|  |  | DR | 6.59 | 0.96 | 7.19 | 0.39 | -38.41 | -32.91 | 5.50 |  |  | 0.001 |  | 1 |
|  |  |  | 7.93 | 0.39 |  |  | -27.40 |  |  |  |  |  |  | 2 |
|  |  |  | 7.04 | 0.51 |  |  | -39.04 |  |  |  |  |  |  | 4 |
| *pnc-1* | 9 | AL | 8.17 | 1.80 | 10.33 | 3.05 |  |  |  |  | 0.69 |  |  | 2 |
|  |  |  | 12.49 | 1.54 |  |  |  |  |  |  |  |  |  | 4 |
|  |  | DR | 7.29 | 1.14 | 7.65 | 0.52 | -10.82 | -23.30 | 17.65 |  | 0.48 | 0.346 |  | 2 |
|  |  |  | 8.02 | 0.93 |  |  | -35.78 |  |  |  |  |  |  | 4 |
|  |  |  |  |  |  |  |  |  |  |  |  |  |  |  |
| N2 | 12 | AL | 7.26 | 0.79 | 9.57 | 2.08 |  |  |  | -13.47 |  |  | 0.573 | 1 |
|  |  |  | 6.34 | 1.11 |  |  |  |  |  |  |  |  |  | 2 |
|  |  |  | 9.12 | 1.08 |  |  |  |  |  |  |  |  |  | 3 |
|  |  |  | 15.56 | 0.90 |  |  |  |  |  |  |  |  |  | 5 |
|  |  | DR | 4.74 | 0.10 | 5.64 | 0.40 | -34.78 | -35.24 | 12.10 | -21.51 |  | 0.113 | 0.042 | 1 |
|  |  |  | 5.88 | 0.45 |  |  | -7.28 |  |  |  |  |  |  | 2 |
|  |  |  | 5.35 | 0.37 |  |  | -41.29 |  |  |  |  |  |  | 3 |
|  |  |  | 6.60 | 0.91 |  |  | -57.59 |  |  |  |  |  |  | 5 |
| *pnc-1* | 12 | AL | 12.19 | 0.07 | 13.74 | 0.36 |  |  |  | 79.50 | 0.36 |  | 0.381 | 2 |
|  |  |  | 13.49 | 0.75 |  |  |  |  |  |  |  |  |  | 5 |
|  |  | DR | 6.34 | 1.38 | 6.43 | 0.13 | -47.99 | -34.87 | 18.56 | -15.94 | 0.12 | 0.185 | 0.746 | 2 |
|  |  |  | 10.55 | 3.55 |  |  | -21.74 |  |  |  |  |  |  | 5 |
|  |  |  |  |  |  |  |  |  |  |  |  |  |  |  |
| N2 | 16 | AL | 9.42 | 1.04 | 7.67 | 0.88 |  |  |  | -30.68 |  |  | 0.020 | 6 |
|  |  |  | 6.73 | 0.75 |  |  |  |  |  |  |  |  |  | 7 |
|  |  |  | 6.85 | 0.92 |  |  |  |  |  |  |  |  |  | 8 |
|  |  | DR | 4.49 | 0.34 | 3.23 | 0.63 | -52.31 | -58.61 | 4.09 | -55.10 |  | 0.015 | 0.006 | 6 |
|  |  |  | 2.71 | 0.35 |  |  | -59.81 |  |  |  |  |  |  | 7 |
|  |  |  | 2.49 | 0.26 |  |  | -63.70 |  |  |  |  |  |  | 8 |
|  |  |  |  |  |  |  |  |  |  |  |  |  |  |  |
|  |  |  |  |  |  |  |  |  |  |  |  |  |  |  |
| *pnc-1* | 16 | AL | 10.25 | 0.62 | 7.35 | 1.70 |  |  |  | -28.82 | 0.88 |  | 0.354 | 6 |
|  |  |  | 7.43 | 0.62 |  |  |  |  |  |  |  |  |  | 7 |
|  |  |  | 4.37 | 0.08 |  |  |  |  |  |  |  |  |  | 8 |
|  | 16 | DR | 5.56 | 0.42 | 3.70 | 0.93 | -45.73 | -47.94 | 10.90 | -51.60 | 0.69 | 0.133 | 0.049 | 6 |
|  |  |  | 2.65 | 0.23 |  |  | -64.33 |  |  |  |  |  |  | 7 |
|  |  |  | 2.90 | 0.73 |  |  | -33.76 |  |  |  |  |  |  | 8 |

All OCR values are normalized to worm number. Mean and SEM was determined for each set of individual replicates, and for replicate experiments. SEM represents standard error of the mean = standard deviation/square root (N-1), where N is the number of replicates. p values were calculated by a two-tailed t-test. Percentage increase vs AL = (mean OCR at given age and OD600 - mean OCR at same age OD600 3.00 performed in parallel)/OCR at same age OD600 3.00 performed in parallel x 100. Experiments with different ID numbers were performed independently of one another. Animals in experiments with the same number (exp #) were hatched, raised, and assayed in parallel at the given age. Animals hatched and raised at the same time, but assayed at different ages, were also given the same experiment number, but noted to have a different age.
