# Supplementary material for: Dietary restriction involves NAD+-dependent mechanisms and a shift toward oxidative metabolism
Source: Aging Cell. 2014 Sep 25;13(6):1075–85. doi: 10.1111/acel.12273 (PMC4244309; doi:10.1111/acel.12273)
Supplement: Supplementary file 8 [file acel0013-1075-sd17.docx]

| Supplemental Table 24. Uncoupled respiration data analyzed per animal shown in Figures 5E, and 5F. | | | | | | | | | | | | | | | | | | | | | | | | | | | | | |
| --- | --- | --- | --- | --- | --- | --- | --- | --- | --- | --- | --- | --- | --- | --- | --- | --- | --- | --- | --- | --- | --- | --- | --- | --- | --- | --- | --- | --- | --- |
|  |  |  |  | |  | |  | |  | |  |  |  | |  | |  |  | |  | |  | |  | |  |  | | |
| **strain** | **age (days)** | **condition** | | **mean OCR/N** | | **SEM** | | **avg** | | **SEM** | **% increase vs. FCCP minus** | **avg % increase vs. FCCP (-)** | | **SEM** | | **avg % increase vs day 9** | ***p value* vs N2** | | ***p value* vs FCCP (-)** | | ***p value* vs AL** | | ***p value* vs day 9** | | ***p value* % change vs AL** | ***p value* % change vs day 9** | | **exp #** |  |
| N2 | 9 | AL | | 11.71 | | 1.39 | | 13.51 | | 1.07 | 9.36 | 21.80 | | 6.97 | |  |  | | 0.09 | |  | |  | |  |  | | 1 |  |
|  |  |  | | 13.38 | | 1.00 | |  | |  | 22.55 |  | |  | |  |  | |  | |  | |  | |  |  | | 2 |  |
|  |  |  | | 15.42 | | 1.46 | |  | |  | 33.49 |  | |  | |  |  | |  | |  | |  | |  |  | | 4 |  |
|  |  | DR | | 10.86 | | 1.73 | | 13.58 | | 1.68 | 64.71 | 87.53 | | 13.11 | |  |  | | 0.02 | | 0.972 | |  | | 0.011 |  | | 1 |  |
|  |  |  | | 16.66 | | 1.05 | |  | |  | 110.13 |  | |  | |  |  | |  | |  | |  | |  |  | | 2 |  |
|  |  |  | | 13.22 | | 0.58 | |  | |  | 87.74 |  | |  | |  |  | |  | |  | |  | |  |  | | 4 |  |
| *pnc-1* | 9 | AL | | 11.44 | | 2.26 | | 15.52 | | 5.78 | 40.01 | 48.50 | | 8.49 | |  | 0.59 | | 0.38 | |  | |  | |  |  | | 2 |  |
|  |  |  | | 19.61 | | 1.46 | |  | |  | 56.98 |  | |  | |  |  | |  | |  | |  | |  |  | | 4 |  |
|  |  | DR | | 20.38 | | 0.78 | | 17.99 | | 5.03 | 179.65 | 129.78 | | 49.87 | |  | 0.31 | | 0.08 | | 0.745 | |  | | 0.249 |  | | 2 |  |
|  |  |  | | 14.43 | | 1.81 | |  | |  | 79.92 |  | |  | |  |  | |  | |  | |  | |  |  | | 4 |  |
|  |  |  | |  | |  | |  | |  |  |  | |  | |  |  | |  | |  | |  | |  |  | |  |  |
| N2 | 12 | AL | | 12.84 | | 0.64 | | 14.03 | | 1.03 | 76.79 | 59.85 | | 22.99 | | 3.86 |  | | 0.10 | |  | | 0.745 | |  | 0.230 | | 1 |  |
|  |  |  | | 13.64 | | 1.93 | |  | |  | 115.07 |  | |  | |  |  | |  | |  | |  | |  |  | | 2 |  |
|  |  |  | | 12.58 | | 0.18 | |  | |  | 38.00 |  | |  | |  |  | |  | |  | |  | |  |  | | 3 |  |
|  |  |  | | 17.05 | | 0.51 | |  | |  | 9.54 |  | |  | |  |  | |  | |  | |  | |  |  | | 5 |  |
|  |  | DR | | 12.19 | | 0.44 | | 10.67 | | 1.19 | 157.30 | 91.98 | | 26.68 | | -21.42 |  | | 0.01 | | 0.078 | | 0.204 | | 0.397 | 0.899 | | 1 |  |
|  |  |  | | 12.35 | | 2.06 | |  | |  | 110.10 |  | |  | |  |  | |  | |  | |  | |  |  | | 2 |  |
|  |  |  | | 7.23 | | 0.65 | |  | |  | 35.00 |  | |  | |  |  | |  | |  | |  | |  |  | | 3 |  |
|  |  |  | | 10.92 | | 1.53 | |  | |  | 65.50 |  | |  | |  |  | |  | |  | |  | |  |  | | 5 |  |
| *pnc-1* | 12 | AL | | 17.51 | | 1.09 | | 15.65 | | 2.62 | 43.58 | 22.96 | | 20.62 | | -12.98 | 0.44 | | 0.29 | |  | | 0.979 | |  | 0.371 | | 2 |  |
|  |  |  | | 13.80 | | 1.53 | |  | |  | 2.34 |  | |  | |  |  | |  | |  | |  | |  |  | | 5 |  |
|  |  | DR | | 11.49 | | 1.18 | | 12.21 | | 1.02 | 81.18 | 51.84 | | 29.34 | | -32.13 | 0.45 | | 0.23 | | 0.225 | | 0.232 | | 0.505 | 0.310 | | 2 |  |
|  |  |  | | 12.93 | | 3.52 | |  | |  | 22.49 |  | |  | |  |  | |  | |  | |  | |  |  | | 5 |  |
|  |  |  | |  | |  | |  | |  |  |  | |  | |  |  | |  | |  | |  | |  |  | |  |  |
| N2 | 16 | AL | | 18.28 | | 1.77 | | 13.18 | | 2.56 | 94.07 | 68.99 | | 12.66 | | -2.44 |  | | 0.11 | |  | | 0.911 | |  | 0.031 | | 6 |  |
|  |  |  | | 10.33 | | 0.75 | |  | |  | 53.45 |  | |  | |  |  | |  | |  | |  | |  |  | | 7 |  |
|  |  |  | | 10.92 | | 1.06 | |  | |  | 59.44 |  | |  | |  |  | |  | |  | |  | |  |  | | 8 |  |
|  |  | DR | | 12.68 | | 0.73 | | 7.01 | | 2.88 | 182.30 | 102.65 | | 46.50 | | -48.36 |  | | 0.27 | | 0.185 | | 0.120 | | 0.523 | 0.770 | | 6 |  |
|  |  |  | | 3.28 | | 0.64 | |  | |  | 21.26 |  | |  | |  |  | |  | |  | |  | |  |  | | 7 |  |
|  |  |  | | 5.08 | | 0.36 | |  | |  | 104.38 |  | |  | |  |  | |  | |  | |  | |  |  | | 8 |  |
| *pnc-1* | 16 | AL | | 16.78 | | 1.04 | | 11.76 | | 3.06 | 63.69 | 57.07 | | 7.48 | | -24.22 | 0.74 | | 0.28 | |  | | 0.506 | |  | 0.511 | | 6 |  |
|  |  |  | | 12.29 | | 0.94 | |  | |  | 65.39 |  | |  | |  |  | |  | |  | |  | |  |  | | 7 |  |
|  |  |  | | 6.22 | | 0.88 | |  | |  | 42.14 |  | |  | |  |  | |  | |  | |  | |  |  | | 8 |  |
|  |  | DR | | 17.73 | | 1.14 | | 9.26 | | 4.29 | 218.63 | 125.88 | | 50.66 | | -48.53 | 0.69 | | 0.27 | | 0.659 | | 0.266 | | 0.250 | 0.962 | | 6 |  |
|  |  |  | | 3.82 | | 0.38 | |  | |  | 44.20 |  | |  | |  |  | |  | |  | |  | |  |  | | 7 |  |
|  |  |  | | 6.22 | | 1.38 | |  | |  | 114.79 |  | |  | |  |  | |  | |  | |  | |  |  | | 8 |  |

Respiration measured after animals were treated with FCCP. All OCR values are normalized to worm number. Data are analyzed and presented similarly to Table S23.
