# Supplementary material for: Dietary restriction involves NAD+-dependent mechanisms and a shift toward oxidative metabolism
Source: Aging Cell. 2014 Sep 25;13(6):1075–85. doi: 10.1111/acel.12273 (PMC4244309; doi:10.1111/acel.12273)
Supplement: Supplementary file 9 [file acel0013-1075-sd18.docx]

| Supplemental Table 25. Respiration under basal conditions analyzed per protein shown in Figures 5B, S3A, and S3B. | | | | | | | | | | | | | | | |
| --- | --- | --- | --- | --- | --- | --- | --- | --- | --- | --- | --- | --- | --- | --- | --- |
|  |  |  |  |  |  |  |  |  |  |  |  |  |  |  |  |
| **strain** | **age (days)** | **condition** | **mean OCR/mg ml^-1^** | **SEM** | **avg** | **SEM** | **% increase vs. AL** | **avg % increase vs. AL** | **SEM** | **avg % increase vs day 9** | ***p* value vs N2** | ***p* value vs AL** | ***p* value vs day 9** | **exp #** |  |
| N2 | 9 | AL | 405.39 | 34.60 | 467.37 | 26.73 |  |  |  |  |  |  |  | 1 |  |
|  |  |  | 526.16 | 72.45 |  |  |  |  |  |  |  |  |  | 2 |  |
|  |  |  | 494.13 | 72.82 |  |  |  |  |  |  |  |  |  | 3 |  |
|  |  |  | 443.78 | 30.01 |  |  |  |  |  |  |  |  |  | 4 |  |
|  |  | DR | 284.13 | 38.16 | 331.23 | 18.93 | -29.91 | -28.68 | 5.50 |  |  | 0.006 |  | 1 |  |
|  |  |  | 330.86 | 33.99 |  |  | -37.12 |  |  |  |  |  |  | 2 |  |
|  |  |  | 333.13 | 55.97 |  |  | -32.58 |  |  |  |  |  |  | 3 |  |
|  |  |  | 376.80 | 25.15 |  |  | -15.09 |  |  |  |  |  |  | 4 |  |
| *pnc-1* | 9 | AL | 270.02 | 53.63 | 415.23 | 86.37 |  |  |  |  | 0.538 |  |  | 2 |  |
|  |  |  | 406.80 | 142.07 |  |  |  |  |  |  |  |  |  | 3 |  |
|  |  |  | 568.87 | 28.95 |  |  |  |  |  |  |  |  |  | 4 |  |
|  |  | DR | 253.65 | 39.19 | 310.13 | 37.95 | -6.06 | -22.16 | 10.03 |  | 0.610 | 0.610 |  | 2 |  |
|  |  |  | 294.46 | 5.58 |  |  | -27.62 |  |  |  |  |  |  | 3 |  |
|  |  |  | 382.28 | 68.08 |  |  | -32.80 |  |  |  |  |  |  | 4 |  |
|  |  |  |  |  |  |  |  |  |  |  |  |  |  |  |  |
| N2 | 12 | AL | 431.70 | 65.74 | 365.21 | 34.93 |  |  |  | -21.86 |  |  | 0.059 | 1 |  |
|  |  |  | 275.76 | 86.14 |  |  |  |  |  |  |  |  |  | 2 |  |
|  |  |  | 407.94 | 34.06 |  |  |  |  |  |  |  |  |  | 3 |  |
|  |  |  | 345.47 | 9.44 |  |  |  |  |  |  |  |  |  | 5 |  |
|  |  | DR | 571.66 | 34.28 | 323.02 | 83.77 | 32.42 | -13.38 | 18.76 | -2.48 |  | 0.658 | 0.927 | 1 |  |
|  |  |  | 239.14 | 27.68 |  |  | -13.28 |  |  |  |  |  |  | 2 |  |
|  |  |  | 270.52 | 48.58 |  |  | -33.69 |  |  |  |  |  |  | 3 |  |
|  |  |  | 210.75 | 40.80 |  |  | -38.99 |  |  |  |  |  |  | 5 |  |
| *pnc-1* | 12 | AL | 433.28 | 56.43 | 410.78 | 22.50 |  |  |  | -1.07 | 0.448 |  | 0.971 | 2 |  |
|  |  |  | 388.28 | 55.29 |  |  |  |  |  |  |  |  |  | 5 |  |
|  |  | DR | 189.20 | 17.52 | 224.01 | 34.82 | -56.33 | -44.84 | 16.26 | -27.77 | 0.481 | 0.046 | 0.218 | 2 |  |
|  |  |  | 258.83 | 78.75 |  |  | -33.34 |  |  |  |  |  |  | 5 |  |
|  |  |  |  |  |  |  |  |  |  |  |  |  |  |  |  |
| N2 | 16 | AL | 530.04 | 53.31 | 615.39 | 64.38 |  |  |  | 31.67 |  |  | 0.064 | 6 |  |
|  |  |  | 741.55 | 104.19 |  |  |  |  |  |  |  |  |  | 7 |  |
|  |  |  | 574.58 | 51.58 |  |  |  |  |  |  |  |  |  | 8 |  |
|  |  | DR | 266.86 | 21.00 | 389.52 | 125.26 | -49.65 | -39.27 | 15.75 | 17.60 |  | 0.184 | 0.610 | 6 |  |
|  |  |  | 640.02 | 86.22 |  |  | -13.69 |  |  |  |  |  |  | 7 |  |
|  |  |  | 261.68 | 23.80 |  |  | -54.46 |  |  |  |  |  |  | 8 |  |
| *pnc-1* | 16 | AL | 496.72 | 44.26 | 599.56 | 184.15 |  |  |  | 44.39 | 0.939 |  | 0.416 | 6 |  |
|  |  |  | 957.25 | 138.62 |  |  |  |  |  |  |  |  |  | 7 |  |
|  |  |  | 344.72 | 26.54 |  |  |  |  |  |  |  |  |  | 8 |  |
|  |  | DR | 503.14 | 44.87 | 451.15 | 93.28 | 1.29 | -19.92 | 14.42 | 45.47 | 0.713 | 0.512 | 0.234 | 6 |  |
|  |  |  | 580.34 | 19.49 |  |  | -39.37 |  |  |  |  |  |  | 7 |  |
|  |  |  | 269.99 | 66.07 |  |  | -21.68 |  |  |  |  |  |  | 8 |  |

All OCR values are normalized to protein content. Data are analyzed and presented similarly to Table S23.
