# Supplementary material for: Dietary restriction involves NAD+-dependent mechanisms and a shift toward oxidative metabolism
Source: Aging Cell. 2014 Sep 25;13(6):1075–85. doi: 10.1111/acel.12273 (PMC4244309; doi:10.1111/acel.12273)
Supplement: Supplementary file 10 [file acel0013-1075-sd19.docx]

| Supplemental Table 26. Uncoupled respiration analyzed per protein shown in Figures S3A, and S3B. | | | | | | | | | | | | | | | | | |
| --- | --- | --- | --- | --- | --- | --- | --- | --- | --- | --- | --- | --- | --- | --- | --- | --- | --- |
|  |  |  |  |  |  |  |  |  |  |  |  |  |  |  |  |  |  |
| **strain** | **age (days)** | **condition** | **mean OCR/mg ml^-1^** | **SEM** | **avg** | **SEM** | **% increase vs. FCCP (-)** | **avg % increase OCR vs. FCCP (-)** | **SEM** | **avg % increase vs day 9** | ***p* value vs N2** | ***p* value vs FCCP (-)** | ***p* value vs AL** | ***p* value vs day 9** | ***p* value % change vs AL** | ***p* value % change vs day 9** | **exp #** |
| N2 | 9 | AL | 473.30 | 56.11 | 598.00 | 41.63 | 16.75 | 27.92 | 5.70 |  |  | 0.039 |  |  |  |  | 1 |
|  |  |  | 645.94 | 72.79 |  |  | 22.76 |  |  |  |  |  |  |  |  |  | 2 |
|  |  |  | 636.57 | 51.08 |  |  | 28.82 |  |  |  |  |  |  |  |  |  | 3 |
|  |  |  | 636.18 | 95.80 |  |  | 43.35 |  |  |  |  |  |  |  |  |  | 4 |
|  |  | DR | 464.50 | 60.89 | 594.07 | 56.64 | 63.48 | 78.69 | 11.01 |  |  | 0.005 | 0.957 |  | 0.006 |  | 1 |
|  |  |  | 688.30 | 57.03 |  |  | 108.03 |  |  |  |  |  |  |  |  |  | 2 |
|  |  |  | 533.62 | 44.05 |  |  | 60.18 |  |  |  |  |  |  |  |  |  | 3 |
|  |  |  | 689.84 | 61.86 |  |  | 83.08 |  |  |  |  |  |  |  |  |  | 4 |
| *pnc-1* | 9 | AL | 394.32 | 79.24 | 573.64 | 130.66 | 46.03 | 38.05 | 9.47 |  | 0.847 | 0.369 |  |  |  |  | 2 |
|  |  |  | 498.69 | 132.09 |  |  | 22.59 |  |  |  |  |  |  |  |  |  | 3 |
|  |  |  | 827.91 | 71.31 |  |  | 45.53 |  |  |  |  |  |  |  |  |  | 4 |
|  |  | DR | 735.32 | 126.94 | 624.52 | 77.98 | 189.89 | 108.21 | 50.22 |  | 0.758 | 0.022 | 0.758 |  | 0.168 |  | 2 |
|  |  |  | 474.08 | 26.06 |  |  | 61.00 |  |  |  |  |  |  |  |  |  | 3 |
|  |  |  | 664.16 | 62.55 |  |  | 73.74 |  |  |  |  |  |  |  |  |  | 4 |
|  |  |  |  |  |  |  |  |  |  |  |  |  |  |  |  |  |  |
| N2 | 12 | AL | 860.77 | 106.28 | 565.56 | 105.15 | 99.39 | 56.52 | 26.17 | -5.42 |  | 0.121 |  | 0.784 |  | 0.327 | 1 |
|  |  |  | 563.25 | 138.17 |  |  | 104.26 |  |  |  |  |  |  |  |  |  | 2 |
|  |  |  | 455.37 | 47.69 |  |  | 11.63 |  |  |  |  |  |  |  |  |  | 3 |
|  |  |  | 382.84 | 20.11 |  |  | 10.82 |  |  |  |  |  |  |  |  |  | 5 |
|  |  | DR | 1485.09 | 150.51 | 673.15 | 272.54 | 159.79 | 91.51 | 26.74 | 13.31 |  | 0.265 | 0.725 | 0.786 | 0.386 | 0.673 | 1 |
|  |  |  | 491.73 | 61.31 |  |  | 105.62 |  |  |  |  |  |  |  |  |  | 2 |
|  |  |  | 371.97 | 82.95 |  |  | 37.50 |  |  |  |  |  |  |  |  |  | 3 |
|  |  |  | 343.81 | 56.29 |  |  | 63.14 |  |  |  |  |  |  |  |  |  | 5 |
| *pnc-1* | 12 | AL | 614.66 | 36.66 | 504.80 | 109.86 | 41.86 | 21.79 | 28.39 | -12.00 | 0.741 | 0.490 |  | 0.738 |  | 0.433 | 2 |
|  |  |  | 394.95 | 59.61 |  |  | 1.72 |  |  |  |  |  |  |  |  |  | 5 |
|  |  | DR | 365.51 | 57.25 | 342.14 | 23.37 | 93.19 | 58.17 | 49.52 | -45.22 | 0.464 | 0.106 | 0.285 | 0.070 | 0.462 | 0.459 | 2 |
|  |  |  | 318.77 | 78.06 |  |  | 23.16 |  |  |  |  |  |  |  |  |  | 5 |

| N2 | 16 | AL | 1034.91 | 61.30 | 1043.53 | 71.91 | 95.25 | 71.35 | 11.98 | 74.50 |  | 0.011 |  | 0.002 |  | 0.016 | 6 |
| --- | --- | --- | --- | --- | --- | --- | --- | --- | --- | --- | --- | --- | --- | --- | --- | --- | --- |
|  |  |  | 1172.17 | 145.73 |  |  | 58.07 |  |  |  |  |  |  |  |  |  | 7 |
|  |  |  | 923.51 | 58.57 |  |  | 60.73 |  |  |  |  |  |  |  |  |  | 8 |
|  |  | DR | 788.08 | 33.71 | 701.84 | 83.63 | 195.31 | 107.31 | 49.96 | 18.14 |  | 0.107 | 0.036 | 0.317 | 0.523 | 0.542 | 6 |
|  |  |  | 782.83 | 171.91 |  |  | 22.31 |  |  |  |  |  |  |  |  |  | 7 |
|  |  |  | 534.61 | 26.00 |  |  | 104.30 |  |  |  |  |  |  |  |  |  | 8 |
| *pnc-1* | 16 | AL | 907.47 | 61.87 | 995.41 | 309.06 | 82.69 | 64.81 | 17.46 | 73.53 | 0.887 | 0.333 |  | 0.277 |  | 0.103 | 6 |
|  |  |  | 1569.24 | 192.41 |  |  | 63.93 |  |  |  |  |  |  |  |  |  | 7 |
|  |  |  | 509.52 | 123.62 |  |  | 47.81 |  |  |  |  |  |  |  |  |  | 8 |
|  |  | DR | 964.07 | 110.29 | 813.92 | 121.25 | 91.61 | 86.64 | 28.75 | 30.33 | 0.489 | 0.077 | 0.614 | 0.259 | 0.324 | 0.651 | 6 |
|  |  |  | 903.75 | 77.16 |  |  | 55.73 |  |  |  |  |  |  |  |  |  | 7 |
|  |  |  | 573.94 | 119.55 |  |  | 112.58 |  |  |  |  |  |  |  |  |  | 8 |

Respiration measured after animals were treated with FCCP. All OCR values are normalized to protein content. Data are analyzed and presented similarly to Table S23.
