# Supplementary material for: Dietary restriction involves NAD+-dependent mechanisms and a shift toward oxidative metabolism
Source: Aging Cell. 2014 Sep 25;13(6):1075–85. doi: 10.1111/acel.12273 (PMC4244309; doi:10.1111/acel.12273)
Supplement: Supplementary file 11 [file acel0013-1075-sd2.docx]

Supplemental Table 1: Comparison of published *C. elegans* liquid DR methods.

| **Liquid DR Method** | **Medium during development** | **AL and DR concentration** **(c.f.u. ml^-1^)** | **Antibiotics** | **DR liquid maintenance** |
| --- | --- | --- | --- | --- |
| Klass^a^ | S-basal | AL = 1x10^10 DR = 1x10^8 | none | 50 worms/solution |
| Vanfleteren^b^ | S-basal, L1 onto NGM plate^τ^ | AL = 1x10^11 DR = 2.4x10^9 | none, 50uM FuDR | Flask with 250mL bacterial solution, took aliquot of 200-500 worms daily to measure survival |
| Dillin^c,d^ | NGM plate^γ^ | AL = 7.5x10^8 DR = 7.5x10^7 | 50ug/ml Carb, 1ug/ml Tet, 10ug/ml Kan, 100ug/ml FuDR | 15 worms into 1ml bacterial solution, put worms into new solution every 3-4 days. |
| Brunetr^e^ | NGM plate | AL = 5x10^11, DR = 5x10^10 | 50ug/ml Amp, 1ug/ml Tet, 10ug/ml Kan, 100mg/L FuDR | 20 worms/ml liquid, worms placed into new bacterial solution every third day |
| Guarente^f^ | NGM plate | AL = 2.5x10^8 DR = 2.5x10^7 | 1mg/ml Eyrthro, 50ug/ml Amp, 12.5ug/ml FuDR, 1mM IPTG | NGM on bottom of each well containing same antibiotics and FuDR as liquid, add fresh bacteria every couple days to maintain bacterial concentration |
| Sinclair, Hart, Blackwell | NGM plate, day 1 adult onto NGM plate with antibiotics | AL = 3x10^9 DR = 3x10^8 | 50ug/ml Amp, 10ug/ml Kan, 1ug/ml Tet, NYS, 100ug/ml FuDR | OP50 arrested in antibiotics at 4C for 1 week prior to dilution. 40 worms/2.5ml solution. Change solution every two weeks. |

Comparison of published *C. elegans* liquid DR methods. Protocols vary with regard to: whether animals develop in liquid or on NGM agar plates, whether and how bacterial growth is arrested, time of placement in DR conditions, the bacterial concentration used for AL and DR conditions, the concentration of animals per volume of solution, and the technical set-up and maintenance required. a. [63]. b [64]. c. [34]. d. [14]. e. [15]. f. [26].
