# Supplementary material for: Dietary restriction involves NAD+-dependent mechanisms and a shift toward oxidative metabolism
Source: Aging Cell. 2014 Sep 25;13(6):1075–85. doi: 10.1111/acel.12273 (PMC4244309; doi:10.1111/acel.12273)
Supplement: Supplementary file 12 [file acel0013-1075-sd20.docx]

| Supplemental Table 27. Respiration per animal normalized to ad-lib conditions shown in Figure 5C. | | | | | | | | | | | | | | | | | | | |
| --- | --- | --- | --- | --- | --- | --- | --- | --- | --- | --- | --- | --- | --- | --- | --- | --- | --- | --- | --- |
|  |  |  |  |  |  |  |  |  |  |  |  |  |  |  |  |  |  |  |  |
| Basal Conditions | | | | | | | | | |  | After addition of FCCP | | | | | | | | |
| **strain** | **age (days)** | **condition** | **Norm. mean OCR** | **Norm. avg** | **SEM** | **Norm. % increase vs day 9** | ***p* value vs AL** | ***p* value vs day 9** | ***p* value vs N2** |  | **Norm. mean OCR** | **Norm. avg** | **SEM** | **Norm. % increase vs day 9** | ***p* value vs N2** | ***p* value vs FCCP (-)** | ***p* value vs AL** | ***p* value vs day 9** | **exp #** |
| N2 | 9 | AL | 1.00 | 1.00 | 0.00 |  |  |  |  |  | 1.09 | 1.22 | 0.09 |  |  | 0.035 |  |  | 1 |
|  |  |  | 1.00 |  |  |  |  |  |  |  | 1.23 |  |  |  |  |  |  |  | 2 |
|  |  |  | 1.00 |  |  |  |  |  |  |  | 1.33 |  |  |  |  |  |  |  | 4 |
|  |  | DR | 0.62 | 0.65 | 0.05 |  | 0.001 |  |  |  | 1.65 | 1.88 | 0.16 |  |  | 0.001 | 0.011 |  | 1 |
|  |  |  | 0.73 |  |  |  |  |  |  |  | 2.10 |  |  |  |  |  |  |  | 2 |
|  |  |  | 0.61 |  |  |  |  |  |  |  | 1.88 |  |  |  |  |  |  |  | 4 |
| *pnc-1* | 9 | AL | 1.00 | 1.00 | 0.00 |  |  |  |  |  | 1.40 | 1.48 | 0.12 |  | 0.094 | 0.029 |  |  | 2 |
|  |  |  | 1.00 |  |  |  |  |  |  |  | 1.57 |  |  |  |  |  |  |  | 4 |
|  |  | DR | 0.89 | 0.77 | 0.18 |  | 0.203 |  | 0.348 |  | 2.80 | 2.30 | 0.71 |  | 0.377 | 0.097 | 0.249 |  | 2 |
|  |  |  | 0.64 |  |  |  |  |  |  |  | 1.80 |  |  |  |  |  |  |  | 4 |
|  |  |  |  |  |  |  |  |  |  |  |  |  |  |  |  |  |  |  |  |
| N2 | 12 | AL | 1.00 | 1.00 | 0.00 |  |  |  |  |  | 1.77 | 1.60 | 0.27 |  |  | 0.041 |  | 0.230 | 1 |
|  |  |  | 1.00 |  |  |  |  |  |  |  | 2.15 |  |  |  |  |  |  |  | 2 |
|  |  |  | 1.00 |  |  |  |  |  |  |  | 1.38 |  |  |  |  |  |  |  | 3 |
|  |  |  | 1.00 |  |  |  |  |  |  |  | 1.10 |  |  |  |  |  |  |  | 5 |
|  |  | DR | 0.65 | 0.65 | 0.12 | -0.44 | 0.036 | 0.983 |  |  | 2.57 | 1.92 | 0.31 | 2.37 |  | 0.004 | 0.397 | 0.899 | 1 |
|  |  |  | 0.93 |  |  |  |  |  |  |  | 2.10 |  |  |  |  |  |  |  | 2 |
|  |  |  | 0.59 |  |  |  |  |  |  |  | 1.35 |  |  |  |  |  |  |  | 3 |
|  |  |  | 0.42 |  |  |  |  |  |  |  | 1.65 |  |  |  |  |  |  |  | 5 |
| *pnc-1* | 12 | AL | 1.00 | 1.00 | 0.00 |  |  |  |  |  | 1.44 | 1.23 | 0.29 |  | 0.372 | 0.381 |  | 0.371 | 3 |
|  |  |  | 1.00 |  |  |  |  |  |  |  | 1.02 |  |  |  |  |  |  |  | 5 |
|  |  | DR | 0.52 | 0.65 | 0.19 | -15.08 | 0.117 | 0.588 | 0.984 |  | 1.81 | 1.52 | 0.41 | -33.92 | 0.412 | 0.114 | 0.505 | 0.310 | 3 |
|  |  |  | 0.78 |  |  |  |  |  |  |  | 1.22 |  |  |  |  |  |  |  | 5 |

| N2 | 16 | AL | 1.00 | 1.00 | 0.00 |  |  |  |  |  | 1.94 | 1.69 | 0.16 |  |  | 0.006 |  | 0.031 | 6 |
| --- | --- | --- | --- | --- | --- | --- | --- | --- | --- | --- | --- | --- | --- | --- | --- | --- | --- | --- | --- |
|  |  |  | 1.00 |  |  |  |  |  |  |  | 1.53 |  |  |  |  |  |  |  | 7 |
|  |  |  | 1.00 |  |  |  |  |  |  |  | 1.59 |  |  |  |  |  |  |  | 8 |
|  |  | DR | 0.48 | 0.41 | 0.02 | -37.59 | 0.000 | 0.009 |  |  | 2.82 | 2.03 | 0.57 | 8.06 |  | 0.026 | 0.523 | 0.770 | 6 |
|  |  |  | 0.40 |  |  |  |  |  |  |  | 1.21 |  |  |  |  |  |  |  | 7 |
|  |  |  | 0.36 |  |  |  |  |  |  |  | 2.04 |  |  |  |  |  |  |  | 8 |
| *pnc-1* | 16 | AL | 1.00 | 1.00 | 0.00 |  |  |  |  |  | 1.64 | 1.57 | 0.09 |  | 0.463 | 0.002 |  | 0.511 | 6 |
|  |  |  | 1.00 |  |  |  |  |  |  |  | 1.65 |  |  |  |  |  |  |  | 7 |
|  |  |  | 1.00 |  |  |  |  |  |  |  | 1.42 |  |  |  |  |  |  |  | 8 |
|  |  | DR | 0.54 | 0.50 | 0.08 | -34.43 | 0.006 | 0.194 | 0.324 |  | 3.19 | 0.99 | 0.30 | -56.79 | 0.752 | 0.028 | 0.250 | 0.962 | 6 |
|  |  |  | 0.36 |  |  |  |  |  |  |  | 1.44 |  |  |  |  |  |  |  | 7 |
|  |  |  | 0.66 |  |  |  |  |  |  |  | 2.15 |  |  |  |  |  |  |  | 8 |

All OCR values are normalized to worm number. Data are analyzed and presented similarly to Table S23. Normalized mean OCR is calculated for each experiment independently. Normalized mean OCR = mean OCR at given condition / mean AL OCR (FCCP(‑)) performed in parallel.
