# Supplementary material for: Dietary restriction involves NAD+-dependent mechanisms and a shift toward oxidative metabolism
Source: Aging Cell. 2014 Sep 25;13(6):1075–85. doi: 10.1111/acel.12273 (PMC4244309; doi:10.1111/acel.12273)
Supplement: Supplementary file 13 [file acel0013-1075-sd21.docx]

| Supplemental Table 28. Respiration per protein normalized to ad-lib conditions shown in Figure 5D. | | | | | | | | | | | | | | | | | | | |
| --- | --- | --- | --- | --- | --- | --- | --- | --- | --- | --- | --- | --- | --- | --- | --- | --- | --- | --- | --- |
|  |  |  |  |  |  |  |  |  |  |  |  |  |  |  |  |  |  |  |  |
| Basal Conditions | | | | | | | | | | | After addition of FCCP | | | | | | | | |
| **strain** | **age (days)** | **condition** | **Norm. mean OCR** | **Norm. avg** | **SEM** | **Norm. % increase vs day 3** | ***p* value vs AL** | ***p* value vs day 9** | ***p* value vs N2** |  | **Norm. mean OCR** | **Norm. avg** | **SEM** | **Norm. % increase vs day 3** | ***p* value vs N2** | ***p* value vs FCCP (-)** | ***p* value vs AL** | ***p* value vs day 9** | **exp #** |
| N2 | 9 | AL | 1.00 | 1.00 | 0.00 |  |  |  |  |  | 1.17 | 1.28 | 0.07 |  |  | 0.003 |  |  | 1 |
|  |  |  | 1.00 |  |  |  |  |  |  |  | 1.23 |  |  |  |  |  |  |  | 2 |
|  |  |  | 1.00 |  |  |  |  |  |  |  | 1.29 |  |  |  |  |  |  |  | 3 |
|  |  |  | 1.00 |  |  |  |  |  |  |  | 1.43 |  |  |  |  |  |  |  | 4 |
|  |  | DR | 0.70 | 0.71 | 0.06 |  | 0.001 |  |  |  | 1.63 | 1.79 | 0.13 |  |  | 0.000 | 0.006 |  | 1 |
|  |  |  | 0.63 |  |  |  |  |  |  |  | 2.08 |  |  |  |  |  |  |  | 2 |
|  |  |  | 0.67 |  |  |  |  |  |  |  | 1.60 |  |  |  |  |  |  |  | 3 |
|  |  |  | 0.85 |  |  |  |  |  |  |  | 1.83 |  |  |  |  |  |  |  | 4 |
| *pnc-1* | 9 | AL | 1.00 | 1.00 | 0.00 |  |  |  |  |  | 1.46 | 1.38 | 0.09 |  | 0.328 | 0.008 |  |  | 2 |
|  |  |  | 1.00 |  |  |  |  |  |  |  | 1.23 |  |  |  |  |  |  |  | 3 |
|  |  |  | 1.00 |  |  |  |  |  |  |  | 1.46 |  |  |  |  |  |  |  | 4 |
|  |  | DR | 0.94 | 0.78 | 0.10 |  | 0.054 |  | 0.496 |  | 2.90 | 2.08 | 0.50 |  | 0.458 | 0.036 | 0.458 |  | 2 |
|  |  |  | 0.72 |  |  |  |  |  |  |  | 1.61 |  |  |  |  |  |  |  | 3 |
|  |  |  | 0.67 |  |  |  |  |  |  |  | 1.74 |  |  |  |  |  |  |  | 4 |
|  |  |  |  |  |  |  |  |  |  |  |  |  |  |  |  |  |  |  |  |
| N2 | 12 | AL | 1.00 | 1.00 | 0.00 |  |  |  |  |  | 1.99 | 1.57 | 0.37 |  |  | 0.074 |  | 0.327 | 1 |
|  |  |  | 1.00 |  |  |  |  |  |  |  | 2.04 |  |  |  |  |  |  |  | 2 |
|  |  |  | 1.00 |  |  |  |  |  |  |  | 1.12 |  |  |  |  |  |  |  | 3 |
|  |  |  | 1.00 |  |  |  |  |  |  |  | 1.11 |  |  |  |  |  |  |  | 5 |
|  |  | DR | 1.32 | 0.87 | 0.23 | 21.44 | 0.441 | 0.401 |  |  | 2.60 | 1.92 | 0.38 | 7.17 |  | 0.015 | 0.386 | 0.673 | 1 |
|  |  |  | 0.87 |  |  |  |  |  |  |  | 2.06 |  |  |  |  |  |  |  | 2 |
|  |  |  | 0.66 |  |  |  |  |  |  |  | 1.38 |  |  |  |  |  |  |  | 3 |
|  |  |  | 0.61 |  |  |  |  |  |  |  | 1.63 |  |  |  |  |  |  |  | 5 |
| *pnc-1* | 12 | AL | 1.00 | 1.00 | 0.00 |  |  |  |  |  | 1.42 | 1.22 | 0.28 |  | 0.446 | 0.391 |  | 0.433 | 2 |
|  |  |  | 1.00 |  |  |  |  |  |  |  | 1.02 |  |  |  |  |  |  |  | 5 |
|  |  | DR | 0.44 | 0.67 | 0.16 | -14.36 | 0.060 | 0.194 | 0.283 |  | 1.93 | 1.58 | 0.50 | -24.03 | 0.504 | 0.108 | 0.462 | 0.459 | 2 |
|  |  |  | 0.67 |  |  |  |  |  |  |  | 1.23 |  |  |  |  |  |  |  | 5 |
|  |  |  |  |  |  |  |  |  |  |  |  |  |  |  |  |  |  |  |  |
|  |  |  |  |  |  |  |  |  |  |  |  |  |  |  |  |  |  |  |  |
|  |  |  |  |  |  |  |  |  |  |  |  |  |  |  |  |  |  |  |  |
|  |  |  |  |  |  |  |  |  |  |  |  |  |  |  |  |  |  |  |  |
| N2 | 16 | AL | 1.00 | 1.00 | 0.00 |  |  |  |  |  | 1.95 | 1.71 | 0.15 |  |  | 0.004 |  | 0.016 | 6 |
|  |  |  | 1.00 |  |  |  |  |  |  |  | 1.58 |  |  |  |  |  |  |  | 7 |
|  |  |  | 1.00 |  |  |  |  |  |  |  | 1.61 |  |  |  |  |  |  |  | 8 |
|  |  | DR | 0.50 | 0.57 | 0.18 | -19.81 | 0.038 | 0.423 |  |  | 2.95 | 2.07 | 0.61 | 16.01 |  | 0.047 | 0.523 | 0.542 | 6 |
|  |  |  | 0.86 |  |  |  |  |  |  |  | 1.22 |  |  |  |  |  |  |  | 7 |
|  |  |  | 0.46 |  |  |  |  |  |  |  | 2.04 |  |  |  |  |  |  |  | 8 |
| *pnc-1* | 16 | AL | 1.00 | 1.00 | 0.00 |  |  |  |  |  | 1.83 | 1.65 | 0.12 |  | 0.698 | 0.003 |  | 0.103 | 6 |
|  |  |  | 1.00 |  |  |  |  |  |  |  | 1.64 |  |  |  |  |  |  |  | 7 |
|  |  |  | 1.00 |  |  |  |  |  |  |  | 1.48 |  |  |  |  |  |  |  | 8 |
|  |  | DR | 1.01 | 0.70 | 0.07 | -10.70 | 0.166 | 0.883 | 0.329 |  | 1.92 | 1.87 | 0.20 | -10.36 | 0.715 | 0.006 | 0.324 | 0.651 | 6 |
|  |  |  | 0.61 |  |  |  |  |  |  |  | 1.56 |  |  |  |  |  |  |  | 7 |
|  |  |  | 0.78 |  |  |  |  |  |  |  | 2.13 |  |  |  |  |  |  |  | 8 |

All OCR values are normalized to protein content. Data are analyzed and presented similarly to Table S27.
