# Supplementary material for: Dietary restriction involves NAD+-dependent mechanisms and a shift toward oxidative metabolism
Source: Aging Cell. 2014 Sep 25;13(6):1075–85. doi: 10.1111/acel.12273 (PMC4244309; doi:10.1111/acel.12273)
Supplement: Supplementary file 14 [file acel0013-1075-sd22.docx]

**SUPPORTING INFORMATION**

**EXPERIMENTAL PROCEDURES**

**Worm strains and maintenance**

All mutants were backcrossed at least 3 times to the wild-type N2 strain that was used for comparison. The strains CF1038 [*daf-16(mu86)*]*,* GA1001 [*aak-2(ok524)*], VC199 [*sir-2.1*(*ok434*)], EU31 [*skn-1*(*zu135*)], PD8120 [*smg-1*(*cc546ts*)], SM190 [*smg-1*(*cc546ts*); *pha-4*(*zu225*)], HA2040 [*sir-2.1(ok434);sir-2.2(n5136);sir-2.4(n5137)*]*,* HA2041 [*sir-2.1(ok434);sir-2.3(ok444);sir-2.4(n5137)*]*,* and *pnc-1 (pk9605)* were used. Strains were maintained and studied at 20°C except for *smg-1*(*cc546ts*); *pha-4*(*zu225*) and *smg-1*(*cc546ts*), which were grown at 25°C to reduce *smg-1* activity, thereby allowing production of PHA-4 protein from the mutant allele (a premature stop) during development (Gaudet & Mango 2002). These animals were shifted to 15°C on the first day of adulthood, partially restoring activity of *smg-1* and leading to the degradation of *pha-4* mRNA in the double mutants. They were then maintained at 15°C for the remainder of their lifespan. Synchronous populations of animals were obtained by hypochlorite treatment of embryos, except for *skn-1* animals, which were synchronized with a 6 hour timed egg-lay.

**DR media preparation**

*Escherichia coli* (OP50) was grown in 500 ml LB in a 1 L flask O/N at 37°C and spun down at 4000 rpm for 10 min. Cells were re-suspended in 500 ml supplemented S basal (1x S-Basal Medium: 0.1 M NaCl, 6 mM K_2_HPO_4_, 44 mM KH_2_PO_4_, H_2_O to 1.0L – autoclaved), 13 μM cholesterol, 0.1 mM Ampicillin, 20.5 μM Kanamycin, 2.25 uM Tetracycline, 23 μM Nystatin, 0.4 μM FUdR), and maintained at room temperature for 7hrs before being placed at 4°C for one week. Immediately prior to transferring animals to liquid cultures, each bacterial culture was allowed to reach room temperature and its optical density (O.D.) at 600nm (A_600_) was determined. The target A_600_ of 3 is outside the linear range of the spectrophotometer; therefore dilutions of each culture were examined to obtain its true A_600_. A starting culture that was dliuted to an A_600_ of 3 was then further diluted to each desired experimental A_600_. To assay for changes in the treated bacteria concentration, 2.5mL treated bacteria at different concentrations were put into a 12-well plate in triplicate, then shaken at 20°C in parallel to lifespan experiments. Ten days later an aliquot was taken from each well, and its concentration was measured using a spectrophotometer.

**Converting A_600_ concentrations to C.F.U.**

A bacterial culture that was analyzed as described above was used to generate the following A_600_ concentrations: 1.5, 1, 0.5, 0.25, and 0.1. These solutions were each further diluted 10^5^, 10^6^, and 10^7^ times with LB, and spread onto 3 NGM plates each. The plates were placed at 37°C overnight. The next day the colonies on each plate were counted, and averaged between replicates. A standard curve was generated with colony forming units (C.F.U.) on the x-axis, and A_600_ on the y-axis. The fitted line was determined and its equation was used to determine the C.F.U. value for each A_600_ concentration of OP50.

**DR Lifespan assays**

Synchronized populations of eggs were hatched and grown at 20°C on standard nematode growth media plates seeded with OP50-1 *E. coli*. Upon day one of adulthood, animals were transferred to NGM agar plates containing 0.1 mM Ampicillin, 20.5 μM Kanamycin, 2.25 μM Tetracycline, 23 μM Nystatin, and 0.8 μM FUdR, and seeded with the cold- and antibiotic- treated OP50 described above. After 48 hours, worms were washed two times in autoclaved M9, and once in supplemented S basal, and transferred to a 12-well tissue culture plate containing 2.5ml cold- and antibiotic- arrested OP50 at different concentrations. 30 worms were put into each well. Plates were kept in a 20°C incubator in a box containing water to prevent liquid from evaporating, on a shaker set to 80 RPM. Animals were transferred to plates containing freshly diluted cold- and antibiotic- arrested OP50 every 2-3 weeks. Animals were scored as dead or alive every other day by gently lifting each worm in the liquid and watching for movement as the worm sunk toward the bottom of the well, or by gently prodding the worm with a pick. Animals that ruptured or died from internal hatching of progeny were excluded from analysis. Lifespans were measured from hatching. Composite lifespans shown for N2, *pnc-1, sir-2.1* and *daf-16* include experiments performed by multiple investigators in the Blackwell, Sinclair and Hart labs, as described in tables S3, S4, S8, S11, S12, S13, S17, and S20. Consistent results were obtained across these experiments.

**Movement assay**

Spontaneous movements, a measure of healthspan (Huang *et al.* 2004), were scored essentially as described (Vrablik *et al.* 2009). An animal was gently placed in a drop of M9 solution on the surface of an unspotted NGM plate, and acclimated for 1 minute at room temperature. Spontaneous thrashes (a maximum bend in one direction) were counted for 30 seconds. Ten animals from each experimental group were examined, with each animal assessed three times. The average number of thrashes that was determined per animal was used to determine a group average with SE.

**Thermotolerance assay**

NGM plates were seeded with an overnight culture of OP50-1, diluted 1:500. Once dry, the plates were used immediately. 20 animals were placed on each plate, with each condition assayed in triplicate. Plates were placed in a 38°C incubator inside a closed box that contained water to keep the plates from drying out. Survival was assessed by gently prodding the worm with a pick hourly until all animals were dead.

**Respiration assay**

Oxygen consumption was measured using the Seahorse XF24 (Seahorse Bioscience, 16 Esquire Rd, North Billerica, MA 01862, USA) essentially as described (Houtkooper *et al.* 2013). Typically, 200 animals were recovered from liquid culture plates with M9 medium, washed three times to eliminate residual bacteria, and resuspended in 500 µl M9 medium. Worms were transferred in 24-well Seahorse plates (50 worms per well) and oxygen consumption was measured 6 times. FCCP treatment was performed at 10 μM final concentration. Following the experiment, plates were frozen at -20°C; upon thawing, the exact worm number was determined for each well. Whole worm lysates were then made from each well using supplemented RIPA buffer. Protein quantification was determined using a BSA protein assay kit, and OCR was normalized to the number of worms or mg/ml of protein.

**Statistical analysis**

Kaplan–Meier survival curves, means, and percentiles were determined using JMP software. P values were calculated using the two-way student t-test. The impact of genotype on lifespan was assessed with one-way ANOVA tests, while the joint impact of genotype and food concentration on lifespan was assessed with two-way ANOVA tests performed in Stata, using mean values obtained from Kaplan–Meier survival curves.

**SUPPLEMENTARY FIGURE LEGENDS**

**Supplementary Figure 1. The NAD^+^ salvage pathway.**

C. elegans synthesize NAD+ through the NAD+ salvage pathway (Vrablik *et al.* 2009). Sirtuins and other NAD+ consumers hydrolyze NAD+, releasing nicotinamide (NAM), an inhibitor of some NAD+ consumers. In invertebrates, nicotinamidases, such as the *C. elegans* PNC-1*,* hydrolyze NAM to produce nicotinic acid (NA). NA is then converted back to NAD through the conserved Preiss-Handler pathway (Vrablik *et al.* 2009).

**Supplementary Figure 2. Analysis of treated bacteria.**

After 10 days at 20°C, treated bacteria diluted in supplemented S basal, containing antibiotics and FuDR, to set concentrations, do not increase in number, but remain alive.

**Supplementary Figure 3. Differential effects of the Mitochondrial Uncoupler FCCP on AL and DR animals.**

Data are all normalized to mg/ml protein. (A,B) The increase in respiration, upon supplementation of 10uM FCCP, is greater in DR animals, than AL. Note that this trend is similar in WT and *pnc-1(pk9605)* animals. One-way ANOVA N2 AL vs DR, p<0.020, two-way ANOVA N2 AL vs DR and age, p<0.0594, one-way ANOVA *pnc-1* AL vs DR, p<0.006, two-way ANOVA *pnc-1* AL vs DR and age, p<0.0180, two-way ANOVA N2 vs *pnc-1* and age, p<0.7009, two-way ANOVA N2 vs *pnc-1* and AL vs DR, p<0.0013 . One-way ANOVA N2 FCCP (-) vs. (+), p<0.0001, two-way ANOVA N2 AL/DR and FCCP (-)/(+), p<0.0005, one-way ANOVA *pnc-1* FCCP (-) vs. (+), p<0.001, two-way ANOVA *pnc-1* AL/DR and FCCP (-)/(+), p<0.0001. In each experiment, samples were assayed in 3-5 replicates per experiment. Wells that did not respond were censored. Individual experimental data, mean, standard error, percent change and statistical analysis are presented in (A,B) Table S25, S26. ** = ttest vs AL, p <0.01; ^@^ = ttest vs age 9, p < 0.055.

**Supplementary Figure 4. Regulation of longevity transcription factors by the IIS and TORC1 pathways.**

The IIS and TOR pathways promote growth, and reductions in each promote lifespan extension (see text). Lifespan extension from IIS reduction is completely dependent upon *daf-16* (Kenyon 2010), which is also required for longevity and stress resistance induced by TORC1 down-regulation (Robida-Stubbs *et al.* 2012). These pathways also inhibit SKN-1, which is involved in longevity and stress resistance deriving from inhibition of each pathway (Tullet *et al.* 2008; Robida-Stubbs *et al.* 2012). PHA-4 also is required for lifespan extension from inhibition of the TOR kinase (Hansen *et al.* 2008; Sheaffer *et al.* 2008).

**ADDITIONAL CITATIONS**

Gaudet J, Mango SE (2002). Regulation of organogenesis by the Caenorhabditis elegans FoxA protein PHA-4. *Science*. **295**, 821-825.

Huang C, Xiong C, Kornfeld K (2004). Measurements of age-related changes of physiological processes that predict lifespan of Caenorhabditis elegans. *Proc Natl Acad Sci U S A*. **101**, 8084-8089.
