# Supplementary material for: Dietary restriction involves NAD+-dependent mechanisms and a shift toward oxidative metabolism
Source: Aging Cell. 2014 Sep 25;13(6):1075–85. doi: 10.1111/acel.12273 (PMC4244309; doi:10.1111/acel.12273)
Supplement: Supplementary file 15 [file acel0013-1075-sd3.docx]

Supplemental Table 2: Bacterial concentration conversion table.

| **A_600_** | **C.F.U. ml^-1^** |
| --- | --- |
| 3* | 3.0E+09 |
| 1.5 | 1.5E+09 |
| 0.5** | 5.0E+08 |
| 0.3 | 3.0E+08 |
| 0.25 | 2.5E+08 |
| 0.15 | 1.5E+08 |
| 0.05 | 5.0E+07 |
| 0.01 | 1.0E+07 |
| 0*** | 0.0E+00 |

A conversion table indicating the C.F.U.ml^-1^ for any given A_600_ of OP50.

*Ad libitum (AL)

**Dietary Restriction (DR)

***Dietary Deprivation (DD)
