# Supplementary material for: Dietary restriction involves NAD+-dependent mechanisms and a shift toward oxidative metabolism
Source: Aging Cell. 2014 Sep 25;13(6):1075–85. doi: 10.1111/acel.12273 (PMC4244309; doi:10.1111/acel.12273)
Supplement: Supplementary file 16 [file acel0013-1075-sd4.docx]

Supplemental Table 3. Statistical analysis of compiled data of wild-type animals on DR shown in Figure 1B and 1C.

| **Strain** | **OD600** | **Average Mean Lifespan** | **SEM** | **Average 75th Percentile** | ***p* value vs AL Mean** | ***p* value vs Preceding OD600 Mean** | **No. Indep. Exper.** |
| --- | --- | --- | --- | --- | --- | --- | --- |
| N2 | 3.00 | 24.9 | <0.1 | 28.5 | NA | NA | 21 |
|  | 1.50 | 29.5 | 0.2 | 33.9 | 0.0031 | NA | 12 |
|  | 0.50 | 39.2 | 0.2 | 45.4 | <0.0001 | <0.0001 | 21 |
|  | 0.30 | 40.3 | 0.3 | 44.4 | <0.0001 | 0.6006 | 5 |
|  | 0.25 | 39.3 | 0.3 | 45.8 | <0.0001 | 0.7109 | 11 |
|  | 0.15 | 39.4 | 0.2 | 42.5 | <0.0001 | 0.9787 | 5 |
|  | 0.05 | 40.2 | 0.2 | 45.7 | <0.0001 | 0.6609 | 6 |
|  | 0.01 | 45.3 | 0.2 | 50.0 | <0.0001 | 0.0154 | 5 |
|  | 0.00 | 43.0 | 0.3 | 49.3 | <0.0001 | 0.4516 | 15 |
|  |  |  |  |  |  |  |  |
|  |  |  |  |  |  |  |  |
|  | **OD600** | **Normalized Mean Lifespan** | **SEM** | **Average % Increase Lifespan vs. AL** | **SEM** | ***p* value vs AL** | ***p* value vs Preceding OD600** |
|  | 3.00 | 1.00 | NA | NA | NA | NA | NA |
|  | 1.50 | 1.17 | 0.04 | <0.1 | <0.1 | <0.0001 | NA |
|  | 0.50 | 1.60 | 0.04 | 59.4 | <0.1 | <0.0001 | <0.0001 |
|  | 0.30 | 1.64 | 0.08 | 64.1 | <0.1 | <0.0001 | 0.6271 |
|  | 0.25 | 1.60 | 0.06 | 60.4 | <0.1 | <0.0001 | 0.7271 |
|  | 0.15 | 1.61 | 0.11 | 61.0 | <0.1 | <0.0001 | 0.9603 |
|  | 0.05 | 1.62 | 0.08 | 61.5 | <0.1 | <0.0001 | 0.9634 |
|  | 0.01 | 1.85 | 0.07 | 84.5 | <0.1 | <0.0001 | 0.0387 |
|  | 0.00 | 1.76 | 0.06 | 76.0 | <0.1 | <0.0001 | 0.4708 |

This is the analysis of all individual experiments in Table S12. Mean and 75th percentiles were determined using JMP software. The 75th percentile refers to the day at which 75% of the population was dead. Normalized mean lifespan is calculated for each experiment independently. Normalized mean lifespan = mean lifespan at given OD600 / mean lifespan at OD600 3.00 performed in parallel. Percentage increase in lifespan = (mean lifespan at given OD600 - mean lifespan at OD600 3.00 performed in parallel)/mean lifespan at OD600 3.00 performed in parallel x 100. SEM represents standard error of the mean = standard deviation/square root (N), where N is the number of observed animals. *p* values were calculated by a two-tailed *t*-test using the average mean lifespan from experiments done on independent days.

Supplemental Table 4. Statistical analysis of compiled data of *daf-16* mutant animals on DR shown in Figure 2A and 2B.

| **Strain** | **OD600** | **Exp Mean Lifespan (days)** | **SEM** | **Exp 75th Percentile (days)** | ***p* value vs AL Mean** | ***p* value Exp. vs N2 Mean** | **N2 Mean Lifespan (days)** | **SEM** | **N2 75th Percentile (days)** | ***p* value vs AL Mean** | **No. Indep. Exper.** |  |
| --- | --- | --- | --- | --- | --- | --- | --- | --- | --- | --- | --- | --- |
| *daf-16* | 3.00 | 23.4 | 0.2 | 27 | NA | 0.2237 | 26.6 | 0.2 | 32 | NA | 3 |  |
|  | 1.50 | 29.0 | 0.3 | 33 | 0.1098 | 0.0854 | 35.9 | 0.3 | 43 | 0.0233 | 3 |  |
|  | 0.50 | 29.5 | 0.3 | 32 | 0.0866 | 0.0143 | 42.0 | 0.3 | 48 | 0.0041 | 3 |  |
|  | 0.30 | 26.5 | 0.3 | 30 | 0.3138 | 0.0171 | 41.12 | 0.4 | 45 | 0.0121 | 3 |  |
|  | 0.15 | 25.8 | 0.1 | 30 | 0.2824 | 0.0021 | 38.9 | 0.2 | 43 | 0.0045 | 3 |  |
|  | 0.05 | 27.4 | <0.1 | 31 | 0.0830 | 0.0001 | 41.0 | 0.1 | 45 | 0.0009 | 3 |  |
|  | 0.01 | 30.0 | 0.3 | 35 | 0.0712 | 0.0037 | 46.4 | 0.2 | 54 | 0.0009 | 3 |  |
|  | 0.00 | 30.9 | 0.4 | 35 | 0.0953 | 0.0472 | 49.2 | 0.4 | 55 | 0.0040 | 2 |  |
|  |  |  |  |  |  |  |  |  |  |  |  |  |
|  |  |  |  |  |  |  |  |  |  |  |  |  |
|  | **OD600** | **Norm. Exp Mean Lifespan (days)** | **SEM** | ***p* value vs AL** | ***p* value vs N2** | **Exp % Increase Lifespan vs AL** | **SEM** | **Norm. N2 Mean Lifespan (days)** | **SEM** | ***p* value vs AL** | **N2 % Lifespan Increase vs AL** | **SEM** |
|  | 3.00 | 1.00 | NA | NA | NA | NA | NA | 1.00 | NA | NA | NA | NA |
|  | 1.50 | 1.24 | 0.02 | 0.0001 | 0.0170 | 24.1 | 0.2 | 1.35 | 0.03 | 0.0001 | 35.1 | 0.3 |
|  | 0.50 | 1.26 | 0.01 | <0.0001 | 0.0002 | 26.3 | 0.2 | 1.58 | 0.03 | <0.0001 | 58.2 | 0.3 |
|  | 0.30 | 1.14 | 0.01 | 0.0001 | 0.0010 | 13.5 | 0.1 | 1.55 | 0.06 | 0.0003 | 54.8 | 0.6 |
|  | 0.15 | 1.11 | 0.06 | 0.0764 | 0.0021 | 11.2 | 0.6 | 1.47 | 0.02 | <0.0001 | 46.5 | 0.2 |
|  | 0.05 | 1.19 | 0.12 | 0.1208 | 0.0351 | 18.7 | 1.3 | 1.55 | 0.08 | 0.0012 | 55.1 | 0.8 |
|  | 0.01 | 1.28 | 0.01 | <0.0001 | 0.0001 | 28.5 | 0.1 | 1.75 | 0.04 | <0.0001 | 75.0 | 0.4 |
|  | 0.00 | 1.39 | 0.01 | <0.0001 | 0.0007 | 39.0 | 0.2 | 1.93 | <0.01 | <0.0001 | 93.4 | <0.1 |

This is the analysis of all individual experiments in Table S13. Experimental animals refer to *daf-16* mutants. The impact of genotype on lifespan was assessed with a one-way ANOVA test, while the joint impact of genotype and food concentration on lifespan was assessed with a two-way ANOVA test performed in STATA, using mean and standard error values obtained from individual Kaplan–Meier survival curves. One-way ANOVA N2/*daf-16* <0.0001; two-way ANOVA N2/*daf-16* and DR <0.0001. The data are analyzed as in Table S3.

Supplemental Table 5. Statistical analysis of compiled data of *skn-1* mutant animals on DR shown in Figure 2C and 2D.

| **Strain** | **OD600** | **Exp Mean Lifespan (days)** | **SEM** | **Exp 75th Percentile (days)** | ***p* value vs AL Mean** | ***p* value Exp. vs N2 Mean** | **N2 Mean Lifespan (days)** | **SEM** | **N2 75th Percentile (days)** | ***p* value vs AL Mean** | **No. Indep. Exper.** |  |
| --- | --- | --- | --- | --- | --- | --- | --- | --- | --- | --- | --- | --- |
| *skn-1* | 3.00 | 25.3 | 0.2 | 30 | NA | 0.9802 | 25.3 | <0.1 | 29 | NA | 6 |  |
|  | 1.50 | 24.5 | 0.1 | 30 | 0.7820 | NA | 26.3 | <0.1 | 33 | 0.1307 | 2 |  |
|  | 0.50 | 29.9 | 0.2 | 34 | 0.0362 | 0.0004 | 37.6 | 0.1 | 45 | <0.0001 | 6 |  |
|  | 0.25 | 28.7 | 0.5 | 30 | 0.2895 | 0.0110 | 42.9 | 0.2 | 52 | <0.0001 | 3 |  |
|  | 0.00 | 27.4 | 0.1 | 30 | 0.3858 | 0.0016 | 48.9 | 0.5 | 57 | <0.0001 | 3 |  |
|  |  |  |  |  |  |  |  |  |  |  |  |  |
|  |  |  |  |  |  |  |  |  |  |  |  |  |
|  | **OD600** | **Norm. Exp Mean Lifespan (days)** | **SEM** | ***p* value vs AL** | ***p* value vs N2** | **Exp % Increase Lifespan vs AL** | **SEM** | **Norm. N2 Mean Lifespan (days)** | **SEM** | ***p* value vs AL** | **N2 % Lifespan Increase vs AL** | **SEM** |
|  | 3.00 | 1.00 | NA | NA | NA | NA | NA | 1.00 | NA | NA | NA | NA |
|  | 1.50 | 1.11 | 0.01 | 0.0133 | 0.3434 | 11.2 | 1.3 | 1.01 | <0.01 | <0.0001 | 1.4 | <0.1 |
|  | 0.50 | 1.21 | 0.01 | 0.0450 | 0.0237 | 20.9 | 1.23 | 1.48 | <0.01 | <0.0001 | 4839 | 0.3 |
|  | 0.25 | 1.20 | 0.04 | 0.2196 | 0.1324 | 20.0 | 3.9 | 1.68 | <0.01 | <0.0001 | 67.6 | 0.3 |
|  | 0.00 | 1.13 | 0.02 | 0.1905 | 0.0116 | 13.3 | 237 | 1.91 | 0.01 | <0.0001 | 91.0 | 1.5 |

This is the analysis of all individual experiments in Table S14. Experimental animals refer to *skn-1* mutants. One-way ANOVA N2/*skn‑1* <0.0001; two-way ANOVA N2/*skn-1* and DR <0.0001. The data are organized and analyzed as in Table S4.

Supplemental Table 6. Statistical analysis of compiled data of *pha-4;smg-1* mutant animals on DR shown in Figure 2E and 2F.

| **Strain** | **OD600** | **Exp Mean Lifespan (days)** | **SEM** | **Exp 75th Percentile (days)** | ***p* value vs AL Mean** | ***p* value Exp. vs *smg-1* Mean** | ***smg-1* Mean Lifespan (days)** | **SEM** | ***smg-1* 75th Percentile (days)** | ***p* value vs AL Mean** | **No. Indep. Exper.** |  |
| --- | --- | --- | --- | --- | --- | --- | --- | --- | --- | --- | --- | --- |
| *pha-4;smg-1* | 3.00 | 23.8 | 0.3 | 27 | NA | 0.42 | 26.6 | 0.3 | 31 | NA | 4 |  |
|  | 1.50 | 21.1 | <0.1 | 24 | 0.42 | NA | NA | NA | NA | NA | 2 |  |
|  | 0.50 | 22.8 | 0.2 | 26 | 0.71 | 0.20 | 33.2 | 1.2 | 41 | 0.51 | 3 |  |
|  | 0.25 | 24.0 | 0.4 | 27 | 0.96 | 0.12 | 34.1 | 1.0 | 41 | 0.36 | 4 |  |
|  | 0.00 | 26.2 | 0.5 | 32 | 0.52 | 0.11 | 38.8 | 1.0 | 48 | 0.22 | 4 |  |
|  |  |  |  |  |  |  |  |  |  |  |  |  |
|  |  |  |  |  |  |  |  |  |  |  |  |  |
|  | **OD600** | **Norm. Exp Mean Lifespan (days)** | **SEM** | ***p* value vs AL** | ***p* value vs *smg-1*** | **Exp % Increase Lifespan vs AL** | **SEM** | **Norm. *smg-1* Mean Lifespan (days)** | **SEM** | ***p* value vs AL** | ***smg-1* % Lifespan Increase vs AL** | **SEM** |
|  | 3.00 | 1.00 | NA | NA | NA | NA | NA | 1.00 | NA | NA | NA | NA |
|  | 1.50 | 1.01 | <0.01 | 0.4064 | 0.0110 | 1.3 | 0.3 | 0.80 | <0.01 | <0.0001 | NA | NA |
|  | 0.50 | 1.04 | <0.01 | 0.1763 | 0.1574 | 3.7 | 0.4 | 1.31 | 0.03 | 0.0623 | 23.2 | 3.5 |
|  | 0.25 | 1.00 | <0.01 | 0.8512 | 0.0256 | 0.4 | 0.3 | 1.27 | 0.02 | 0.0214 | 27.3 | 2.5 |
|  | 0.00 | 1.10 | 0.01 | 0.1954 | 0.0242 | 9.7 | 1.0 | 1.45 | 0.02 | 0.0035 | 44.6 | 2.6 |

This is the analysis of all individual experiments in Table S15. Experimental animals refer to *pha‑4;smg-1* mutants. *smg-1* mutants are the control. One-way ANOVA *smg‑1/pha-4;smg-1* <0.0001; two-way ANOVA *smg-1/pha-4;smg-1* and DR 0.0004. Experimental conditions can be found under “worm strains and maintenance” in the section, “experimental procedures”. Data are organized and analyzed as in Table S4.

Supplemental Table 7. Statistical analysis of compiled data of *aak-2* mutant animals on DR shown in Figure 2G and 2H.

| **Strain** | **OD600** | **Exp Mean Lifespan (days)** | **SEM** | **Exp 75th Percentile (days)** | ***p* value vs AL Mean** | ***p* value Exp. vs N2 Mean** | **N2 Mean Lifespan (days)** | **SEM** | **N2 75th Percentile (days)** | ***p* value vs AL Mean** | **No. Indep. Exper.** |  |
| --- | --- | --- | --- | --- | --- | --- | --- | --- | --- | --- | --- | --- |
| *aak-2* | 3.00 | 20.1 | < 0.1 | 23 | NA | 0.1487 | 22.9 | 0.2 | 26 | NA | 2 |  |
|  | 0.50 | 23.0 | 0.4 | 24 | 0.4168 | 0.0954 | 37.4 | 0.7 | 46 | 0.0695 | 2 |  |
|  | 0.25 | 23.3 | 0.1 | 29 | 0.0530 | 0.0150 | 37.7 | 0.3 | 45 | 0.0182 | 2 |  |
|  | 0.05 | 25.6 | <0.1 | 29 | 0.0002 | 0.0517 | 37.7 | 0.6 | 44 | 0.0421 | 2 |  |
|  | 0.00 | 26.1 | 0.2 | 29 | 0.0345 | 0.0114 | 38.1 | 0.1 | 43 | 0.0082 | 2 |  |
|  |  |  |  |  |  |  |  |  |  |  |  |  |
|  |  |  |  |  |  |  |  |  |  |  |  |  |
|  | **OD600** | **Norm. Exp Mean Lifespan (days)** | **SEM** | ***p* value vs AL** | ***p* value vs N2** | **Exp % Increase Lifespan vs AL** | **SEM** | **Norm. N2 Mean Lifespan (days)** | **SEM** | ***p* value vs AL** | **N2 % Lifespan Increase vs AL** | **SEM** |
|  | 3.00 | 1.00 | NA | NA | NA | NA | NA | 1.00 | NA | NA | NA | NA |
|  | 0.50 | 1.14 | 0.02 | 0.4050 | 0.2278 | 14.5 | 2.0 | 1.64 | 0.05 | 0.1279 | 64.4 | 4.7 |
|  | 0.25 | 1.16 | 0.01 | 0.0407 | 0.0942 | 16.2 | 0.6 | 1.65 | 0.03 | 0.0544 | 65.3 | 2.6 |
|  | 0.05 | 1.27 | <0.01 | 0.0004 | 0.2161 | 27.2 | 0.1 | 1.65 | 0.04 | 0.0925 | 65.5 | 4.2 |
|  | 0.00 | 1.30 | 0.01 | 0.0408 | 0.0542 | 29.9 | 1.1 | 1.66 | 0.01 | 0.0089 | 66.4 | 1.1 |

This is the analysis of all individual experiments in Table S16. Experimental animals refer to *aak-2* mutants. One-way ANOVA N2/*aak‑2* <0.0001; two‑way ANOVA N2/*aak-2* and DR <0.0001.The data are organized and analyzed as in Table S4.

Supplemental Table 8. Statistical analysis of compiled data of *sir-2.1* mutant animals on DR shown in in Figure 3A and 3B.

| **Strain** | **OD600** | **Exp Mean Lifespan (days)** | **SEM** | **Exp 75th Percentile (days)** | ***p* value vs AL Mean** | ***p* value Exp. vs N2 Mean** | **N2 Mean Lifespan (days)** | **SEM** | **N2 75th Percentile (days)** | ***p* value vs AL Mean** | **No. Indep. Exper.** |  |
| --- | --- | --- | --- | --- | --- | --- | --- | --- | --- | --- | --- | --- |
| *sir-2.1* | 3.00 | 27.4 | 0.1 | 31 | NA | 0.0313 | 23.9 | 0.2 | 28 | NA | 4 |  |
|  | 1.50 | 33.9 | 0.3 | 39 | 0.0656 | 0.2926 | 28.6 | 0.5 | 32 | 0.2641 | 4 |  |
|  | 0.50 | 38.4 | 0.1 | 42 | <0.0001 | 0.0911 | 40.3 | 0.1 | 45 | <0.0001 | 4 |  |
|  | 0.30 | 36.8 | 0.1 | 40 | <0.0001 | 0.4713 | 37.8 | 0.1 | 42 | 0.0003 | 4 |  |
|  | 0.15 | 35.5 | 0.1 | 39 | 0.0007 | 0.0558 | 40.7 | 0.3 | 43 | 0.0003 | 4 |  |
|  | 0.05 | 33.1 | 0.3 | 38 | 0.0325 | 0.1020 | 40.2 | < 0.1 | 46 | 0.0006 | 2 |  |
|  | 0.01 | 35.3 | 0.2 | 38 | 0.0089 | 0.0329 | 43.0 | 0.3 | 48 | 0.0001 | 4 |  |
|  | 0.00 | 40.4 | 0.4 | 46 | 0.0079 | 0.2914 | 45.4 | 0.4 | 51 | 0.0003 | 4 |  |
|  |  |  |  |  |  |  |  |  |  |  |  |  |
|  |  |  |  |  |  |  |  |  |  |  |  |  |
|  | **OD600** | **Norm. Exp Mean Lifespan (days)** | **SEM** | ***p* value vs AL** | ***p* value vs N2** | **Exp % Increase Lifespan vs AL** | **SEM** | **Norm. N2 Mean Lifespan (days)** | **SEM** | ***p* value vs AL** | **N2 % Lifespan Increase vs AL** | **SEM** |
|  | 3.00 | 1.00 | NA | NA | 0.6448 | NA | NA | 1.00 | NA | NA | NA | NA |
|  | 1.50 | 1.24 | 0.01 | 0.0568 | 0.7111 | 23.9 | 1.1 | 1.18 | 0.01 | 0.1170 | 18.4 | 1.4 |
|  | 0.50 | 1.40 | <0.01 | 0.0000 | 0.0143 | 40.5 | 0.5 | 1.69 | 0.01 | 0.0001 | 69.5 | 1.2 |
|  | 0.30 | 1.34 | 0.01 | 0.0004 | 0.0339 | 34.5 | 0.6 | 1.55 | 0.01 | 0.0001 | 55.4 | 0.8 |
|  | 0.15 | 1.30 | <0.01 | 0.0005 | 0.0270 | 29.6 | 0.5 | 1.72 | 0.02 | 0.0020 | 71.7 | 2.4 |
|  | 0.05 | 1.17 | 0.01 | 0.0045 | 0.0643 | 17.3 | 0.7 | 1.59 | 0.01 | 0.0006 | 58.5 | 1.2 |
|  | 0.01 | 1.29 | 0.01 | 0.0034 | 0.0011 | 28.9 | 0.7 | 1.80 | 0.01 | 0.0000 | 80.0 | 1.0 |
|  | 0.00 | 1.47 | 0.01 | 0.0046 | 0.0126 | 47.4 | 1.3 | 1.89 | 0.01 | 0.0000 | 89.4 | 0.8 |

This is the analysis of all individual experiments in Table S17. Experimental animals refer to *sir-2.1* mutants. One-way ANOVA N2/*sir‑2.1* <0.0880; two‑way ANOVA N2/*sir-2.1* and DR <0.0001.The data are organized and analyzed as in Table S4.

Supplemental Table 9. Statistical analysis of compiled data of *sir-2.1;sir-2.2;sir-2.4* mutant animals on DR shown in Figure 3C and 3D.

| **Strain** | **OD600** | **Exp Mean Lifespan (days)** | **SEM** | **Exp 75th Percentile (days)** | ***p* value vs AL Mean** | ***p* value Exp. vs N2 Mean** | **N2 Mean Lifespan (days)** | **SEM** | **N2 75th Percentile (days)** | ***p* value vs AL Mean** | **No. Indep. Exper.** |  |
| --- | --- | --- | --- | --- | --- | --- | --- | --- | --- | --- | --- | --- |
| *sir-2.1;sir-2.2;sir-2.4* | 3.00 | 26.7 | 0.3 | 29 | NA | 0.3159 | 23.3 | 0.3 | 27 | NA | 2 |  |
|  | 0.50 | 30.5 | 0.1 | 37 | 0.2240 | 0.0951 | 38.7 | 0.5 | 46 | 0.0364 | 2 |  |
|  | 0.25 | 33.6 | 0.8 | 44 | 0.3234 | 0.3265 | 40.0 | 0.1 | 48 | 0.0107 | 2 |  |
|  | 0.05 | 31.7 | 1.0 | 38 | 0.4518 | 0.1876 | 41.8 | 0.2 | 46 | 0.0118 | 2 |  |
|  | 0.00 | 36.7 | 1.2 | 43 | 0.2996 | 0.6722 | 40.4 | 0.5 | 46 | 0.0363 | 2 |  |
|  |  |  |  |  |  |  |  |  |  |  |  |  |
|  |  |  |  |  |  |  |  |  |  |  |  |  |
|  | **OD600** | **Norm. Exp Mean Lifespan (days)** | **SEM** | ***p* value vs AL** | ***p* value vs N2** | **Exp % Increase Lifespan vs AL** | **SEM** | **Norm. N2 Mean Lifespan (days)** | **SEM** | ***p* value vs AL** | **N2 % Lifespan Increase vs AL** | **SEM** |
|  | 3.00 | 1.00 | NA | NA | 0.4226 | NA | NA | 1.00 | NA | NA | NA | NA |
|  | 0.50 | 1.15 | 0.01 | 0.1078 | 0.1489 | 14.7 | 0.7 | 1.68 | 0.04 | 0.0949 | 67.6 | 4.0 |
|  | 0.25 | 1.25 | 0.01 | 0.1082 | 0.0644 | 25.2 | 1.4 | 1.72 | 0.01 | 0.0143 | 72.4 | 1.5 |
|  | 0.05 | 1.18 | 0.02 | 0.2097 | 0.0355 | 18.0 | 1.9 | 1.80 | 0.01 | 0.0073 | 80.0 | 1.2 |
|  | 0.00 | 1.36 | 0.03 | 0.1473 | 0.1433 | 36.4 | 2.7 | 1.73 | <0.01 | 0.0001 | 73.5 | 0.1 |

This is the analysis of all individual experiments in Table S18. Experimental animals refer to *sir‑2.1;sir-2.2;sir-2.4* mutants. One-way ANOVA N2/*sir-2.1;sir-2.2;sir-2.4* <0.0270; two-way ANOVA N2/ *sir-2.1;sir-2.2;sir-2.4* and DR equals 0.0001.The data are organized and analyzed as in Table S4.

Supplemental Table 10. Statistical analysis of compiled data of *sir-2.1;sir-2.3;sir-2.4* mutant animals on DR shown in Figure 3E and 3F.

| **Strain** | **OD600** | **Exp Mean Lifespan (days)** | **SEM** | **Exp 75th Percentile (days)** | ***p* value vs AL Mean** | ***p* value Exp. vs N2 Mean** | **N2 Mean Lifespan (days)** | **SEM** | **N2 75th Percentile (days)** | ***p* value vs AL Mean** | **No. Indep. Exper.** |  |
| --- | --- | --- | --- | --- | --- | --- | --- | --- | --- | --- | --- | --- |
| *sir-2.1;sir-2.3;sir-2.4* | 3.00 | 23.6 | 0.1 | 26 | NA | 0.2631 | 22.0 | 0.1 | 24 | NA | 2 |  |
|  | 0.50 | 36.1 | 0.1 | 41 | 0.0116 | 0.0318 | 41.5 | 0.1 | 46 | 0.0004 | 2 |  |
|  | 0.30 | 35.8 | < 0.1 | 43 | 0.0064 | 0.1965 | 38.1 | 0.2 | 45 | 0.0059 | 2 |  |
|  | 0.05 | 35.7 | 0.1 | 41 | 0.0064 | 0.0783 | 42.2 | 0.4 | 42 | 0.0070 | 2 |  |
|  | 0.00 | 38.3 | 0.5 | 41 | 0.1079 | 0.9804 | 38.2 | 0.1 | 43 | 0.0022 | 2 |  |
|  |  |  |  |  |  |  |  |  |  |  |  |  |
|  |  |  |  |  |  |  |  |  |  |  |  |  |
|  | **OD600** | **Norm. Exp Mean Lifespan (days)** | **SEM** | ***p* value vs AL** | ***p* value vs N2** | **Exp % Increase Lifespan vs AL** | **SEM** | **Norm. N2 Mean Lifespan (days)** | **SEM** | ***p* value vs AL** | **N2 % Lifespan Increase vs AL** | **SEM** |
|  | 3.00 | 1.00 | NA | NA | NA | NA | NA | 1.00 | NA | NA | NA | NA |
|  | 0.50 | 1.53 | <0.01 | 0.0020 | 0.0057 | 53.2 | 0.3 | 1.89 | <0.01 | 0.0002 | 88.7 | 0.2 |
|  | 0.30 | 1.52 | <0.01 | 0.0146 | 0.1705 | 52.0 | 0.4 | 1.73 | 0.01 | 0.0113 | 73.3 | 1.5 |
|  | 0.05 | 1.51 | <0.01 | 0.0015 | 0.0170 | 51.3 | 0.2 | 1.92 | 0.01 | 0.0029 | 91.9 | 1.1 |
|  | 0.00 | 1.62 | 0.02 | 0.6341 | 0.5256 | 61.8 | 1.6 | 1.73 | <0.01 | 0.0096 | 73.5 | 0.2 |

This is the analysis of all individual experiments in Table S19. Experimental animals refer to *sir‑2.1;sir-2.3;sir-2.4* mutants. One-way ANOVA N2/*sir-2.1;sir-2.3;sir-2.4* equals 0.089; two-way ANOVA N2/*sir-2.1;sir-2.3;sir-2.4* and DR  <0.0001.The data are organized and analyzed as in Table S4.

Supplemental Table 11. Statistical analysis of compiled data of *pnc-1* mutant animals on DR shown in Figure 3G and 3H.

| **Strain** | **OD600** | **Exp Mean Lifespan (days)** | **SEM** | **Exp 75th Percentile (days)** | ***p* value vs AL Mean** | ***p* value Exp. vs N2 Mean** | **N2 Mean Lifespan (days)** | **SEM** | **N2 75th Percentile (days)** | ***p* value vs AL Mean** | **No. Indep. Exper.** |  |
| --- | --- | --- | --- | --- | --- | --- | --- | --- | --- | --- | --- | --- |
| *pnc-1* | 3.00 | 21.5 | 0.1 | 25 | NA | 0.0020 | 25.3 | 0.2 | 29 | NA | 9 |  |
|  | 1.50 | 24.3 | 0.3 | 28 | 0.0352 | 0.4284 | 31.6 | 1.1 | 35 | 0.1162 | 2 |  |
|  | 0.50 | 28.6 | 0.2 | 36 | <0.0001 | <0.0001 | 38.6 | 0.2 | 45 | <0.0001 | 10 |  |
|  | 0.30 | 26.9 | 0.2 | 34 | 0.0007 | 0.0037 | 44.1 | 0.3 | 48 | 0.0001 | 3 |  |
|  | 0.15 | 22.6 | 0.4 | 29 | 0.3476 | 0.0410 | 38.9 | 0.4 | 44 | 0.0008 | 2 |  |
|  | 0.05 | 24.6 | 0.4 | 33 | 0.0969 | 0.0008 | 43.9 | 0.3 | 46 | <0.0001 | 4 |  |
|  | 0.00 | 23.9 | 0.4 | 32 | 0.1980 | 0.0318 | 38.3 | 0.5 | 45 | 0.0006 | 3 |  |
|  |  |  |  |  |  |  |  |  |  |  |  |  |
|  |  |  |  |  |  |  |  |  |  |  |  |  |
|  | **OD600** | **Norm. Exp Mean Lifespan (days)** | **SEM** | ***p* value vs AL** | ***p* value vs N2** | **Exp % Increase Lifespan vs AL** | **SEM** | **Norm. N2 Mean Lifespan (days)** | **SEM** | ***p* value vs AL** | **N2 % Lifespan Increase vs AL** | **SEM** |
|  | 3.00 | 1.00 | NA | NA | NA | NA | NA | 1.00 | NA | NA | NA | NA |
|  | 1.50 | 1.15 | 0.01 | 0.0080 | 0.5863 | 14.6 | 1.4 | 1.24 | 0.02 | 0.0004 | 24.1 | 1.7 |
|  | 0.50 | 1.27 | 0.01 | <0.0001 | 0.0037 | 32.1 | 0.9 | 1.58 | 0.01 | <0.0001 | 57.2 | 1.0 |
|  | 0.30 | 1.23 | 0.01 | 0.0002 | 0.0064 | 27.0 | 1.2 | 1.79 | 0.01 | <0.0001 | 77.2 | 1.6 |
|  | 0.15 | 1.07 | 0.02 | 0.1636 | 0.0931 | 6.6 | 1.8 | 1.56 | 0.02 | <0.0001 | 56.1 | 2.1 |
|  | 0.05 | 1.08 | 0.02 | 0.0593 | 0.1073 | 14.8 | 2.0 | 1.68 | 0.02 | <0.0001 | 76.5 | 1.7 |
|  | 0.00 | 1.15 | 0.02 | 0.1177 | 0.0248 | 15.1 | 1.9 | 1.82 | 0.02 | <0.0001 | 82.0 | 1.9 |

This is the analysis of all individual experiments in Table S20. Experimental animals refer to *pnc-1* mutants. One-way ANOVA N2/*pnc‑1* <0.0001; two‑way ANOVA N2/*pnc-1* and DR <0.0001.The data are organized and analyzed as in Table S4.
