# Supplementary material for: Dietary restriction involves NAD+-dependent mechanisms and a shift toward oxidative metabolism
Source: Aging Cell. 2014 Sep 25;13(6):1075–85. doi: 10.1111/acel.12273 (PMC4244309; doi:10.1111/acel.12273)
Supplement: Supplementary file 17 [file acel0013-1075-sd5.docx]

| Supplemental Table 12. Analyses of individual DR experiments performed on wild-type animals shown in Figure 1B and 1C. | | | | | | | |
| --- | --- | --- | --- | --- | --- | --- | --- |
|  |  |  |  |  |  |  |  |
| **Strain** | **OD600** | **Mean Lifespan (days)** | **Normalized Mean Lifespan (days)** | **75th Percentile (days)** | **# Animals** | **% increase Lifespan vs. AL** | **ID** |
| N2 | 3.00 | 28.8 | 1.00 | 33 | 57 | 0.0 | 1a* |
|  | 1.50 | 38.7 | 1.34 | 47 | 50 | 34.4 | 1a* |
|  | 0.50 | 46.1 | 1.60 | 52 | 57 | 60.2 | 1a* |
|  | 0.30 | 47.0 | 1.63 | 52 | 61 | 63.5 | 1a* |
|  | 0.15 | 41.4 | 1.44 | 47 | 45 | 43.9 | 1a* |
|  | 0.05 | 42.6 | 1.48 | 45 | 52 | 48.1 | 1a* |
|  | 0.01 | 48.9 | 1.70 | 54 | 52 | 70.0 | 1a* |
|  |  |  |  |  |  |  |  |
|  | 3.00 | 27.0 | 1.00 | 32 | 64 | 0.0 | 1b* |
|  | 1.50 | 37.5 | 1.39 | 42 | 72 | 39.3 | 1b* |
|  | 0.50 | 41.4 | 1.54 | 45 | 63 | 53.6 | 1b* |
|  | 0.30 | 39.7 | 1.47 | 42 | 66 | 47.3 | 1b* |
|  | 0.15 | 39.3 | 1.46 | 42 | 59 | 46.0 | 1b* |
|  | 0.05 | 40.1 | 1.49 | 45 | 86 | 48.7 | 1b* |
|  | 0.01 | 47.1 | 1.75 | 55 | 78 | 74.9 | 1b* |
|  | 0.00 | 52.1 | 1.93 | 57 | 77 | 93.4 | 1b* |
|  |  |  |  |  |  |  |  |
|  | 3.00 | 24.0 | 1.00 | 31 | 55 | 0.0 | 1c* |
|  | 1.50 | 31.5 | 1.32 | 40 | 61 | 31.6 | 1c* |
|  | 0.50 | 38.5 | 1.61 | 46 | 57 | 60.8 | 1c* |
|  | 0.30 | 36.8 | 1.53 | 40 | 63 | 53.5 | 1c* |
|  | 0.15 | 35.9 | 1.50 | 40 | 56 | 49.7 | 1c* |
|  | 0.05 | 40.3 | 1.68 | 46 | 53 | 68.3 | 1c* |
|  | 0.01 | 43.1 | 1.80 | 53 | 55 | 80.0 | 1c* |
|  | 0.00 | 46.4 | 1.93 | 53 | 56 | 93.4 | 1c* |
|  |  |  |  |  |  |  |  |
|  | 3.00 | 21.6 | 1.00 | 24 | 18 | 0.0 | 1d |
|  | 1.50 | 24.6 | 1.14 | 23 | 27 | 13.8 | 1d |
|  | 0.50 | 37.1 | 1.72 | 44 | 17 | 71.7 | 1d |
|  | 0.30 | 41.2 | 1.91 | 45 | 40 | 90.9 | 1d |
|  | 0.15 | 36.3 | 1.68 | 40 | 22 | 68.2 | 1d |
|  | 0.01 | 43.4 | 2.01 | 45 | 36 | 100.9 | 1d |
|  | 0.00 | 44.0 | 2.04 | 52 | 29 | 103.8 | 1d |
|  |  |  |  |  |  |  |  |
|  | 3.00 | 22.3 | 1.00 | 23 | 31 | 0.0 | 1e |
|  | 1.50 | 23.2 | 1.04 | 25 | 63 | 4.0 | 1e |
|  | 0.50 | 41.8 | 1.87 | 44 | 36 | 87.4 | 1e |
|  | 0.30 | 36.9 | 1.65 | 44 | 16 | 65.4 | 1e |
|  | 0.15 | 43.9 | 1.97 | 44 | 14 | 97.1 | 1e |
|  | 0.01 | 43.9 | 1.97 | 44 | 17 | 96.9 | 1e |
|  | 0.00 | 38.8 | 1.74 | 44 | 25 | 74.2 | 1e |
|  |  |  |  |  |  |  |  |
|  | 3.00 | 19.7 | 1.00 | 21 | 29 | 0.0 | 1f |
|  | 1.50 | 20.8 | 1.05 | 21 | 32 | 5.4 | 1f |
|  | 0.50 | 34.0 | 1.73 | 41 | 34 | 72.5 | 1f |
|  | 0.25 | 31.8 | 1.62 | 37 | 43 | 61.6 | 1f |
|  | 0.00 | 33.4 | 1.70 | 41 | 40 | 69.6 | 1f |
|  |  |  |  |  |  |  |  |
|  | 3.00 | 21.7 | 1.00 | 25 | 35 | 0.0 | 1g |
|  | 0.50 | 41.2 | 1.90 | 48 | 28 | 90.0 | 1g |
|  | 0.25 | 39.3 | 1.81 | 46 | 42 | 81.1 | 1g |
|  | 0.05 | 40.5 | 1.87 | 42 | 28 | 86.9 | 1g |
|  | 0.00 | 37.5 | 1.73 | 42 | 33 | 72.7 | 1g |
|  |  |  |  |  |  |  |  |
|  | 3.00 | 24.9 | 1.00 | 29 | 31 | 0.0 | 1h |
|  | 0.50 | 36.1 | 1.45 | 44 | 34 | 45.1 | 1h |
|  | 0.25 | 40.7 | 1.64 | 50 | 30 | 63.6 | 1h |
|  | 0.05 | 43.1 | 1.73 | 50 | 34 | 73.1 | 1h |
|  | 0.00 | 43.4 | 1.74 | 50 | 37 | 74.3 | 1h |
|  |  |  |  |  |  |  |  |
|  | 3.00 | 24.2 | 1.00 | 26 | 37 | 0.0 | 1i |
|  | 0.50 | 33.6 | 1.39 | 43 | 31 | 38.9 | 1i |
|  | 0.25 | 36.1 | 1.49 | 43 | 35 | 49.4 | 1i |
|  | 0.05 | 34.8 | 1.44 | 46 | 25 | 44.0 | 1i |
|  | 0.00 | 38.7 | 1.60 | 43 | 30 | 60.1 | 1i |
|  |  |  |  |  |  |  |  |
|  | 3.00 | 23.7 | 1.00 | 25 | 22 | 0.0 | 1j |
|  | 0.50 | 31.3 | 1.32 | 33 | 31 | 31.9 | 1j |
|  | 0.25 | 29.2 | 1.23 | 33 | 34 | 23.2 | 1j |
|  | 0.00 | 33.3 | 1.40 | 36 | 18 | 40.2 | 1j |
|  |  |  |  |  |  |  |  |
|  | 3.00 | 25.9 | 1.00 | 31 | 32 | 0.0 | 1k |
|  | 1.50 | 26.3 | 1.01 | 33 | 36 | 1.4 | 1k |
|  | 0.50 | 39.4 | 1.52 | 46 | 35 | 52.1 | 1k |
|  | 0.25 | 43.9 | 1.70 | 53 | 37 | 69.6 | 1k |
|  | 0.00 | 51.7 | 1.99 | 60 | 31 | 99.4 | 1k |
|  |  |  |  |  |  |  |  |
|  | 3.00 | 25.9 | 1.00 | 31 | 32 | 0.0 | 1l |
|  | 1.50 | 26.3 | 1.01 | 33 | 36 | 1.4 | 1l |
|  | 0.50 | 39.4 | 1.52 | 46 | 35 | 52.1 | 1l |
|  | 0.25 | 43.9 | 1.70 | 53 | 37 | 69.6 | 1l |
|  | 0.00 | 51.7 | 1.99 | 60 | 31 | 99.4 | 1l |
|  |  |  |  |  |  |  |  |
|  | 3.00 | 24.9 | 1.00 | 28 | 31 | 0.0 | 1m |
|  | 0.50 | 43.6 | 1.75 | 53 | 58 | 74.9 | 1m |
|  |  |  |  |  |  |  |  |
|  | 3.00 | 22.6 | 1.00 | 25 | 35 | 0.0 | 1n |
|  | 1.50 | 29.3 | 1.30 | 33 | 34 | 29.8 | 1n |
|  | 0.50 | 43.8 | 1.94 | 49 | 28 | 94.0 | 1n |
|  | 0.25 | 42.5 | 1.88 | 48 | 33 | 88.2 | 1n |
|  | 0.00 | 45.1 | 2.00 | 53 | 39 | 99.7 | 1n |
|  |  |  |  |  |  |  |  |
|  | 3.00 | 25.2 | 1.00 | 28 | 30 | 0.0 | 1o |
|  | 0.50 | 42.7 | 1.69 | 46 | 56 | 69.3 | 1o |
|  |  |  |  |  |  |  |  |
|  | 3.00 | 27.9 | 1.00 | 31 | 25 | 0.0 | 1p |
|  | 1.50 | 31.8 | 1.14 | 38 | 35 | 14.3 | 1p |
|  | 0.50 | 38.3 | 1.37 | 46 | 44 | 37.3 | 1p |
|  | 0.25 | 36.5 | 1.31 | 41 | 35 | 31.2 | 1p |
|  | 0.00 | 34.7 | 1.25 | 41 | 36 | 24.7 | 1p |
|  |  |  |  |  |  |  |  |
|  | 3.00 | 21.6 | 1.00 | 31 | 23 | 0.0 | 1q |
|  | 3.00 | 27.2 | 1.00 | 31 | 35 | 0.0 | 1q |
|  | 3.00 | 26.8 | 1.00 | 31 | 38 | 0.0 | 1q |
|  | 0.50 | 40.0 | 1.85 | 45 | 37 | 85.5 | 1q |
|  | 0.50 | 38.1 | 1.40 | 42 | 27 | 40.3 | 1q |
|  | 0.50 | 36.9 | 1.38 | 45 | 30 | 37.6 | 1q |
|  |  |  |  |  |  |  |  |
|  | 3.00 | 25.0 | 1.00 | 27 | 54 | 0.0 | 1s |
|  | 1.50 | 26.9 | 1.08 | 32 | 38 | 7.7 | 1s |
|  | 0.50 | 32.7 | 1.31 | 43 | 30 | 30.7 | 1s |
|  | 0.25 | 44.2 | 1.77 | 50 | 36 | 76.9 | 1s |
|  | 0.00 | 50.3 | 2.01 | 62 | 32 | 101.3 | 1s |
|  |  |  |  |  |  |  |  |
|  | 3.00 | 29.4 | 1.00 | 34 | 77 | 0.0 | 1t |
|  | 1.50 | 37.2 | 1.27 | 41 | 53 | 26.7 | 1t |
|  | 0.50 | 47.2 | 1.61 | 53 | 49 | 60.6 | 1t |
|  | 0.25 | 44.1 | 1.50 | 50 | 59 | 50.2 | 1t |
|  | 0.00 | 42.6 | 1.45 | 50 | 26 | 45.1 | 1t |
| ID* indicates experiments performed in the Sinclair/Hart lab. All others were performed in the Blackwell Lab. | | | | | | | |
|  |  |  |  |  |  |  |  |
| Experiments with different ID numbers were performed independently of one another. Number of animals represents number of observed deaths. The data are analyzed as in Table S3. | | | | | | | |
