# Supplementary material for: Dietary restriction involves NAD+-dependent mechanisms and a shift toward oxidative metabolism
Source: Aging Cell. 2014 Sep 25;13(6):1075–85. doi: 10.1111/acel.12273 (PMC4244309; doi:10.1111/acel.12273)
Supplement: Supplementary file 18 [file acel0013-1075-sd6.docx]

| Supplemental Table 13. Analyses of individual DR experiments performed on *daf-16* animals shown in Figure 2A and 2B. | | | | | | | | | | | | | |
| --- | --- | --- | --- | --- | --- | --- | --- | --- | --- | --- | --- | --- | --- |
|  |  |  |  |  |  |  |  |  |  |  |  |  |  |
| **Strain** | **OD600** | **Exp Mean Lifespan (days)** | **Normalized Exp Mean Lifespan (days)** | **Exp 75th Percentile (days)** | **# Exp Animals** | **% increase lifespan vs AL** | **N2 Mean Lifespan (days)** | **Normalized N2 Mean Lifespan (days)** | **N2 75th Percentile (days)** | **# N2 animals** | **N2 % lifespan increase vs AL** | **Exp vs N2 % increase lifespan** | **ID** |
| *daf-16* | 3.00 | 25.5 | 1.00 | 28 | 68 | 0.0 | 28.8 | 1.00 | 33 | 57 | 0.0 | -11.3 | 3a* |
|  | 1.50 | 30.9 | 1.21 | 33 | 69 | 21.1 | 38.7 | 1.34 | 47 | 50 | 34.4 | -20.1 | 3a* |
|  | 0.50 | 31.7 | 1.24 | 33 | 50 | 24.2 | 46.1 | 1.60 | 52 | 57 | 60.2 | -31.2 | 3a* |
|  | 0.30 | 29.4 | 1.15 | 31 | 67 | 15.2 | 47.0 | 1.63 | 52 | 61 | 63.5 | -37.5 | 3a* |
|  | 0.15 | 27.0 | 1.06 | 31 | 67 | 6.0 | 41.4 | 1.44 | 47 | 45 | 43.9 | -34.7 | 3a* |
|  | 0.05 | 27.0 | 1.06 | 31 | 48 | 5.8 | 42.6 | 1.48 | 45 | 52 | 48.1 | -36.6 | 3a* |
|  | 0.01 | 32.5 | 1.27 | 38 | 63 | 27.5 | 48.9 | 1.70 | 54 | 52 | 70.0 | -33.5 | 3a* |
|  |  |  |  |  |  |  |  |  |  |  |  |  |  |
|  | 3.00 | 24.6 | 1.00 | 28 | 59 | 0.0 | 27.0 | 1.00 | 32 | 64 | 0.0 | -8.6 | 3b* |
|  | 1.50 | 31.2 | 1.27 | 35 | 61 | 26.9 | 37.5 | 1.39 | 42 | 72 | 39.3 | -16.8 | 3b* |
|  | 0.50 | 31.4 | 1.27 | 35 | 43 | 27.4 | 41.4 | 1.54 | 45 | 63 | 53.6 | -24.2 | 3b* |
|  | 0.30 | 27.9 | 1.13 | 32 | 42 | 13.1 | 39.7 | 1.47 | 42 | 66 | 47.3 | -29.9 | 3b* |
|  | 0.15 | 26.3 | 1.07 | 32 | 55 | 6.9 | 39.3 | 1.46 | 42 | 59 | 46.0 | -33.1 | 3b* |
|  | 0.05 | 27.8 | 1.13 | 32 | 62 | 13.0 | 40.1 | 1.49 | 45 | 86 | 48.7 | -30.6 | 3b* |
|  | 0.01 | 31.5 | 1.28 | 35 | 61 | 28.1 | 47.1 | 1.75 | 55 | 78 | 74.9 | -33.1 | 3b* |
|  | 0.00 | 33.9 | 1.38 | 38 | 57 | 37.6 | 52.1 | 1.93 | 57 | 77 | 93.4 | -35.0 | 3b* |
|  |  |  |  |  |  |  |  |  |  |  |  |  |  |
|  | 3.00 | 19.9 | 1.00 | 24 | 46 | 0.0 | 24.0 | 1.00 | 31 | 55 | 0.0 | -16.9 | 3c* |
|  | 1.50 | 24.7 | 1.24 | 30 | 48 | 24.3 | 31.5 | 1.32 | 40 | 61 | 31.6 | -21.6 | 3c* |
|  | 0.50 | 25.3 | 1.27 | 28 | 49 | 27.2 | 38.5 | 1.61 | 46 | 57 | 60.8 | -34.3 | 3c* |
|  | 0.30 | 22.3 | 1.12 | 28 | 54 | 12.2 | 36.8 | 1.53 | 40 | 63 | 53.5 | -39.3 | 3c* |
|  | 0.15 | 24.0 | 1.21 | 28 | 44 | 20.6 | 35.9 | 1.50 | 40 | 56 | 49.7 | -33.1 | 3c* |
|  | 0.05 | 27.3 | 1.37 | 31 | 51 | 37.3 | 40.3 | 1.68 | 46 | 53 | 68.3 | -32.2 | 3c* |
|  | 0.01 | 25.8 | 1.30 | 31 | 51 | 29.8 | 43.1 | 1.80 | 53 | 55 | 80.0 | -40.1 | 3c* |
|  | 0.00 | 28.0 | 1.40 | 31 | 56 | 40.5 | 46.4 | 1.93 | 53 | 56 | 93.4 | -39.7 | 3c* |
| ID* indicates experiments performed in the Sinclair/Hart lab. | | | | |  |  |  |  |  |  |  |  |  |
|  |  |  |  |  |  |  |  |  |  |  |  |  |  |

Experimental animals refer to *daf-16* mutants. Data was analyzed as in Table S12.
