# Supplementary material for: Dietary restriction involves NAD+-dependent mechanisms and a shift toward oxidative metabolism
Source: Aging Cell. 2014 Sep 25;13(6):1075–85. doi: 10.1111/acel.12273 (PMC4244309; doi:10.1111/acel.12273)
Supplement: Supplementary file 19 [file acel0013-1075-sd7.docx]

| Supplemental Table 14. Analyses of individual DR experiments performed on *skn-1* animals shown in Figure 2C and 2D. | | | | | | | | | | | | | |
| --- | --- | --- | --- | --- | --- | --- | --- | --- | --- | --- | --- | --- | --- |
|  |  |  |  |  |  |  |  |  |  |  |  |  |  |
| **Strain** | **OD600** | **Exp Mean Lifespan (days)** | **Normalized Exp Mean Lifespan (days)** | **Exp 75th Percentile (days)** | **# Exp Animals** | **% increase lifespan vs AL** | **N2 Mean Lifespan (days)** | **Normalized N2 Mean Lifespan (days)** | **N2 75th Percentile (days)** | **# N2 animals** | **N2 % lifespan increase vs AL** | **Exp vs N2 % increase lifespan** | **ID** |
| *skn-1* | 3.00 | 30.5 | 1.00 | 35 | 41 | 0.0 | 24.9 | 1.00 | 29 | 31 | 0.0 | 22.8 | 6a |
|  | 0.50 | 27.8 | 0.91 | 30 | 36 | -8.9 | 36.1 | 1.45 | 44 | 34 | 45.1 | -22.9 | 6a |
|  | 0.25 | 28.3 | 0.93 | 30 | 33 | -7.3 | 40.7 | 1.64 | 50 | 30 | 63.6 | -30.5 | 6a |
|  | 0.00 | 27.2 | 0.89 | 30 | 54 | -10.9 | 43.4 | 1.74 | 50 | 37 | 74.3 | -37.3 | 6a |
|  |  |  |  |  |  |  |  |  |  |  |  |  |  |
|  | 3.00 | 26.6 | 1.00 | 27 | 31 | 0.0 | 24.2 | 1.00 | 26 | 37 | 0.0 | 10.3 | 6b |
|  | 0.50 | 27.6 | 1.04 | 30 | 29 | 3.7 | 33.6 | 1.39 | 43 | 31 | 38.9 | -17.7 | 6b |
|  |  |  |  |  |  |  |  |  |  |  |  |  |  |
|  | 3.00 | 20.0 | 1.00 | 25 | 31 | 0.0 | 25.9 | 1.00 | 31 | 32 | 0.0 | -22.9 | 6d |
|  | 1.50 | 23.8 | 1.19 | 29 | 42 | 19.1 | 26.3 | 1.01 | 33 | 36 | 1.4 | -9.5 | 6d |
|  | 0.50 | 31.2 | 1.56 | 37 | 39 | 56.0 | 39.4 | 1.52 | 46 | 35 | 52.1 | -20.9 | 6d |
|  | 0.25 | 34.0 | 1.70 | 35 | 52 | 70.3 | 43.9 | 1.70 | 53 | 37 | 69.6 | -22.6 | 6d |
|  | 0.00 | 28.4 | 1.42 | 33 | 42 | 42.3 | 51.7 | 1.99 | 60 | 31 | 99.4 | -45.0 | 6d |
|  |  |  |  |  |  |  |  |  |  |  |  |  |  |
|  | 3.00 | 24.4 | 1.00 | 32 | 34 | 0.0 | 25.9 | 1.00 | 31 | 32 | 0.0 | -5.8 | 6e |
|  | 1.50 | 25.2 | 1.03 | 32 | 30 | 3.2 | 26.3 | 1.01 | 33 | 36 | 1.4 | -4.1 | 6e |
|  | 0.50 | 26.8 | 1.10 | 32 | 33 | 9.6 | 39.4 | 1.52 | 46 | 35 | 52.1 | -32.1 | 6e |
|  | 0.25 | 23.7 | 0.97 | 26 | 40 | -2.9 | 43.9 | 1.70 | 53 | 37 | 69.6 | -46.1 | 6e |
|  | 0.00 | 26.5 | 1.09 | 28 | 44 | 8.5 | 51.7 | 1.99 | 60 | 31 | 99.4 | -48.8 | 6e |
|  |  |  |  |  |  |  |  |  |  |  |  |  |  |
|  | 3.00 | 22.9 | 1.00 | 25 | 78 | 0.0 | 25.9 | 1.00 | 28 | 74 | 0.0 | -11.7 | 6f |
|  | 0.50 | 32.7 | 1.43 | 36 | 108 | 42.8 | 38.8 | 1.49 | 45 | 76 | 49.5 | -15.6 | 6f |
|  |  |  |  |  |  |  |  |  |  |  |  |  |  |
|  | 3.00 | 27.3 | 1.00 | 34 | 133 | 0.0 | 25.2 | 1.00 | 31 | 96 | 0.0 | 8.3 | 6g |
|  | 0.50 | 33.3 | 1.22 | 39 | 118 | 22.1 | 38.4 | 1.52 | 44 | 94 | 52.2 | -13.2 | 6g |
|  |  |  |  |  |  |  |  |  |  |  |  |  |  |

Experimental animals refer to *skn-1* mutants. Data are organized and analyzed as in Table S12.
