# Supplementary material for: Dietary restriction involves NAD+-dependent mechanisms and a shift toward oxidative metabolism
Source: Aging Cell. 2014 Sep 25;13(6):1075–85. doi: 10.1111/acel.12273 (PMC4244309; doi:10.1111/acel.12273)
Supplement: Supplementary file 20 [file acel0013-1075-sd8.docx]

| Supplemental Table 15. Analyses of individual DR experiments performed on *pha-4;smg-1* animals shown in Figure 2E and 2F. | | | | | | | | | | | | | |
| --- | --- | --- | --- | --- | --- | --- | --- | --- | --- | --- | --- | --- | --- |
|  |  |  |  |  |  |  |  |  |  |  |  |  |  |
| **Strain** | **OD600** | **Exp Mean Lifespan (days)** | **Normalized Exp Mean Lifespan (days)** | **Exp 75th Percentile (days)** | **# Exp Animals** | **% increase lifespan vs AL** | ***smg-1* Mean Lifespan (days)** | **Normalized *smg-1* Mean Lifespan (days)** | ***smg-1* 75th Percentile (days)** | **# *smg-1* animals** | ***smg-1* % lifespan increase vs AL** | **Exp vs *smg-1* % increase lifespan** | **ID** |
| *pha-4;smg-1* | 3.00 | 29.1 | 1.00 | 31 | 0.00 | 29.0 | 25.0 | 1.00 | 25 | 43 | 0.0 | 16.6 | 11a |
|  | 0.25 | 29.9 | 1.03 | 31 | 2.75 | 31.0 | 28.0 | 1.12 | 31 | 42 | 12.0 | 6.9 | 11a |
|  | 0.00 | 32.0 | 1.10 | 34 | 9.81 | 36.0 | 32.0 | 1.28 | 39 | 52 | 28.0 | 0.0 | 11a |
|  |  |  |  |  |  |  |  |  |  |  |  |  |  |
|  | 3.00 | 24.4 | 1.00 | 25 | 0.00 | 23.0 | 25.0 | 1.00 | 25 | 43 | 0.0 | -2.6 | 11b |
|  | 0.50 | 25.0 | 1.02 | 25 | 2.46 | 38.0 | 25.0 | 1.00 | 31 | 41 | 0.0 | -0.2 | 11b |
|  | 0.25 | 25.3 | 1.04 | 25 | 3.74 | 39.0 | 28.0 | 1.12 | 31 | 42 | 12.0 | -9.8 | 11b |
|  | 0.00 | 30.6 | 1.26 | 40 | 25.50 | 36.0 | 32.0 | 1.28 | 39 | 52 | 28.0 | -4.5 | 11b |
|  |  |  |  |  |  |  |  |  |  |  |  |  |  |
|  | 3.00 | 20.4 | 1.00 | 24 | 0.00 | 39.0 | 28.2 | 1.00 | 36 | 34 | 0.0 | -27.9 | 11c |
|  | 1.50 | 21.1 | 1.04 | 24 | 3.51 | 41.0 | 22.6 | 0.80 | 32 | 39 | -19.8 | -6.9 | 11c |
|  | 0.50 | 22.2 | 1.09 | 26 | 8.91 | 52.0 | 40.8 | 1.46 | 50 | 49 | 44.7 | -45.7 | 11c |
|  | 0.25 | 20.6 | 1.01 | 26 | 1.13 | 56.0 | 39.0 | 1.43 | 50 | 34 | 38.3 | -47.2 | 11c |
|  | 0.00 | 22.5 | 1.10 | 32 | 10.36 | 47.0 | 43.6 | 1.61 | 55 | 33 | 54.5 | -48.5 | 11c |
|  |  |  |  |  |  |  |  |  |  |  |  |  |  |
|  | 3.00 | 21.4 | 1.00 | 28 | 0.00 | 39.0 | 28.2 | 1.00 | 36 | 34 | 0.0 | -24.1 | 11d |
|  | 1.50 | 21.2 | 0.99 | 24 | -0.95 | 44.0 | 22.6 | 0.80 | 32 | 39 | -19.8 | -6.3 | 11d |
|  | 0.50 | 21.3 | 1.00 | 26 | -0.43 | 54.0 | 40.8 | 1.46 | 50 | 49 | 44.7 | -47.8 | 11d |
|  | 0.25 | 20.1 | 0.94 | 24 | -5.90 | 49.0 | 39.0 | 1.43 | 50 | 34 | 38.3 | -48.4 | 11d |
|  | 0.00 | 19.9 | 0.93 | 22 | -6.99 | 45.0 | 43.6 | 1.61 | 55 | 33 | 54.5 | -54.3 | 11d |
|  |  |  |  |  |  |  |  |  |  |  |  |  |  |

Data are organized and analyzed as in Table S12. *smg-1* mutants are the control.
