# Supplementary material for: Dietary restriction involves NAD+-dependent mechanisms and a shift toward oxidative metabolism
Source: Aging Cell. 2014 Sep 25;13(6):1075–85. doi: 10.1111/acel.12273 (PMC4244309; doi:10.1111/acel.12273)
Supplement: Supplementary file 21 [file acel0013-1075-sd9.docx]

| Supplemental Table 16. Analyses of individual DR experiments performed on *aak-2* animals shown in Figure 2G and 2H. | | | | | | | | | | | | | |
| --- | --- | --- | --- | --- | --- | --- | --- | --- | --- | --- | --- | --- | --- |
|  |  |  |  |  |  |  |  |  |  |  |  |  |  |
| **Strain** | **OD600** | **Exp Mean Lifespan (days)** | **Normalized Exp Mean Lifespan (days)** | **Exp 75th Percentile (days)** | **# Exp Animals** | **% increase lifespan vs AL** | **N2 Mean Lifespan (days)** | **Normalized N2 Mean Lifespan (days)** | **N2 75th Percentile (days)** | **# N2 animals** | **N2 % lifespan increase vs AL** | **Exp vs N2 % increase lifespan** | **ID** |
| *aak-2* | 3.00 | 20.2 | 1.00 | 26 | 54 | 0.0 | 21.7 | 1.00 | 25 | 35 | 0.0 | -7.0 | 2a |
|  | 0.50 | 25.9 | 1.28 | 27 | 51 | 28.3 | 41.2 | 1.90 | 48 | 28 | 90.0 | -37.2 | 2a |
|  | 0.25 | 24.1 | 1.20 | 31 | 27 | 19.5 | 39.3 | 1.81 | 46 | 42 | 81.1 | -38.6 | 2a |
|  | 0.05 | 25.5 | 1.27 | 31 | 24 | 26.6 | 40.5 | 1.87 | 42 | 28 | 86.9 | -37.0 | 2a |
|  | 0.00 | 24.9 | 1.24 | 28 | 32 | 23.6 | 37.5 | 1.73 | 42 | 33 | 72.7 | -33.4 | 2a |
|  |  |  |  |  |  |  |  |  |  |  |  |  |  |
|  | 3.00 | 20.0 | 1.00 | 20 | 34 | 0.0 | 24.2 | 1.00 | 26 | 37 | 0.0 | -17.2 | 2b |
|  | 0.50 | 20.1 | 1.01 | 20 | 45 | 0.6 | 33.6 | 1.39 | 43 | 31 | 38.9 | -40.0 | 2b |
|  | 0.25 | 22.6 | 1.13 | 26 | 34 | 12.8 | 36.1 | 1.49 | 43 | 35.00 | 49.4 | -37.5 | 2b |
|  | 0.05 | 25.6 | 1.28 | 26 | 38 | 27.8 | 34.8 | 1.44 | 46 | 25.00 | 44.0 | -26.6 | 2b |
|  | 0.00 | 27.2 | 1.36 | 30 | 32 | 36.1 | 38.7 | 1.60 | 43 | 30 | 60.1 | -29.6 | 2b |

Experimental animals refer to *aak-2* mutants. Data are organized and analyzed as in Table S12.
